# Gender-specific accuracy of lipid accumulation product index for the screening of metabolic syndrome in general adults: a meta-analysis and comparative analysis with other adiposity indicators

**DOI:** 10.1186/s12944-024-02190-1

**Published:** 2024-06-26

**Authors:** Bendix Samarta Witarto, Andro Pramana Witarto, Visuddho Visuddho, Citrawati Dyah Kencono Wungu, Ummi Maimunah, Purwo Sri Rejeki, Delvac Oceandy

**Affiliations:** 1 Medical Program, Faculty of Medicine, Universitas Airlangga, Surabaya, Indonesia; 2 Division of Biochemistry, Department of Medical Physiology and Biochemistry, Faculty of Medicine, Universitas Airlangga, Jl. Mayjen Prof. Dr. Moestopo 47, Surabaya, East Java 60132 Indonesia; 3 Institute of Tropical Disease, Universitas Airlangga, Surabaya, Indonesia; 4 Division of Gastroenterology and Hepatology, Department of Internal Medicine, Dr. Soetomo General Hospital, Faculty of Medicine, Universitas Airlangga, Surabaya, Indonesia; 5 Division of Physiology, Department of Medical Physiology and Biochemistry, Faculty of Medicine, Universitas Airlangga, Surabaya, Indonesia; 6 Division of Cardiovascular Science, Manchester Academic Health Science Centre, University of Manchester, Manchester, M13 9PG UK

**Keywords:** Lipid accumulation product, Diagnostic test accuracy, Meta-analysis, Metabolic syndrome, Non-communicable disease, Screening

## Abstract

**Background:**

Lipid accumulation product (LAP) is a novel predictor index of central lipid accumulation associated with metabolic and cardiovascular diseases. This study aims to investigate the accuracy of LAP for the screening of metabolic syndrome (MetS) in general adult males and females and its comparison with other lipid-related indicators.

**Methods:**

A systematic literature search was conducted in PubMed, Scopus, Web of Science, Cumulative Index to Nursing and Allied Health Literature (CINAHL), and ProQuest for eligible studies up to May 8, 2024. Outcomes were pooled mean difference (MD), odds ratio (OR), and diagnostic accuracy parameters (sensitivity, specificity, and area under the summary receiver operating characteristic [AUSROC] curve). Comparative analysis was conducted using *Z*-test.

**Results:**

Forty-three studies involving 202,313 participants (98,164 males and 104,149 females) were included. Pooled MD analysis showed that LAP was 45.92 (*P* < 0.001) and 41.70 units (*P* < 0.001) higher in men and women with MetS, respectively. LAP was also significantly associated with MetS, with pooled ORs of 1.07 (*P* < 0.001) in men and 1.08 (*P* < 0.001) in women. In men, LAP could detect MetS with a pooled sensitivity of 85% (95% CI: 82%–87%), specificity of 81% (95% CI: 80%–83%), and AUSROC curve of 0.88 (95% CI: 0.85–0.90), while in women, LAP had a sensitivity of 83% (95% CI: 80%–86%), specificity of 80% (95% CI: 78%–82%), and AUSROC curve of 0.88 (95% CI: 0.85–0.91). LAP had a significantly higher AUSROC curve (*P* < 0.05) for detecting MetS compared to body mass index (BMI), waist-to-height ratio (WHtR), waist-to-hip ratio (WHR), body roundness index (BRI), a body shape index (ABSI), body adiposity index (BAI), conicity index (CI) in both genders, and waist circumference (WC) and abdominal volume index (AVI) in females.

**Conclusion:**

LAP may serve as a simple, cost-effective, and more accurate screening tool for MetS in general adult male and female populations.

**Supplementary Information:**

The online version contains supplementary material available at 10.1186/s12944-024-02190-1.

## Introduction

Metabolic syndrome (MetS) refers to a group of metabolic disorders characterized by central obesity, impaired glucose control, elevated triglyceride levels, decreased levels of high-density lipoprotein-cholesterol (HDL-C), elevated blood pressure, and insulin resistance [1]. MetS is becoming a serious health problem and economic burden, as its global prevalence is high and remains growing in both developed and developing countries [2, 3]. A recent meta-analysis in 2022 estimated that the global prevalence of MetS varied from 12.5% to 31.4% depending on the diagnostic criteria, with Americas and Eastern Mediterranean as regions with the highest prevalence across different MetS definitions [4]. According to a survey conducted among adults in the United States, the prevalence of MetS has increased with a significant trend from 37.6% in 2011–2012 to 41.8% in 2017–2018 [5].

MetS has been associated with two times higher risk of cardiovascular and cerebrovascular diseases and 1.5 times higher mortality rate [6]. Additionally, given its close association with insulin resistance, a person with MetS is at a high risk of developing type 2 diabetes mellitus, which is known as a potentially debilitating chronic disease with various macrovascular and microvascular complications, including coronary artery disease, stroke, and diabetic kidney disease [7, 8]. These consequences further highlight the importance of early and accurate identification of high-risk individuals with MetS to prevent any adverse outcomes related to its development. However, the current diagnostic criteria for MetS are complex to conduct as they include many metabolic components, making early detection of individuals with MetS difficult. Hence, it would be more convenient to use a simpler, rapid, and inexpensive indicator with high accuracy for the screening of MetS, mainly in a large population [9].

In 2005, Kahn [10] proposed a novel, safe and practical index for reflecting excess central lipid accumulation among adults, based on a combination of two economical measurements, namely waist circumference (WC) and concentration of triglyceride (TG) measured in the fasting state. This index was later called ‘lipid accumulation product’ (LAP). It is calculated as [WC (cm) – 65] × [TG (mmol/L)] for men, and [WC (cm) – 58] × [TG (mmol/L)] for women. LAP is closely associated with insulin resistance and has been linked to various metabolic and cardiovascular risk factors [11, 12]. Previous studies in different men and women populations have shown that LAP has a high predictive performance and outperforms other adiposity indicators, such as WC, body mass index (BMI), waist-to-height ratio (WHtR), waist-to-hip ratio (WHR), and visceral adiposity index (VAI), at detecting MetS. However, the accuracies reported still varied between studies [13–15]. Moreover, a study by Endukuru et al. [16] has shown contradictory findings, where LAP was found to have a lower power than several indicators in identifying MetS. Although LAP has commonly been the focus of earlier studies, there are no reviews to date that have demonstrated evidence regarding the conclusive accuracy of LAP and whether it is arguable to use LAP for the screening of MetS. Thus, this systematic review and meta-analysis aim to explore the screening performance of LAP as a detection tool for MetS in general male and female adults, and its comparison with other adiposity indicators.

## Materials and methods

This systematic review and meta-analysis were conducted in conformity with the Preferred Reporting Items for Systematic Reviews and Meta-Analyses (PRISMA) 2020 guidelines [17] (see Supplementary Table 1 in Additional file 1 for the completed PRISMA 2020 checklist of this study) and guided by Cochrane Handbook for Systematic Reviews of Diagnostic Test Accuracy Version 2.0 [18]. The detailed protocol of this study has been registered on the International Prospective Register of Systematic Reviews (PROSPERO; https://www.crd.york.ac.uk/prospero/; registration number: CRD42021259797).

### Search strategy and study selection process

A computerized literature searching was conducted in PubMed, Scopus, Web of Science, Cumulative Index to Nursing and Allied Health Literature (CINAHL) via EBSCO, and ProQuest from inception to May 8, 2024. The Medical Subject Headings (MeSH) and other free-text keywords were applied to formulate the following full search term: ("lipid accumulation product*" OR "LAP") AND ("metabolic syndrome" OR "MetS" OR "MetSyn" OR "syndrome X" OR "metabolic X syndrome" OR "insulin resistance syndrome" OR "cardiometabolic syndrome" OR "metabolic cardiovascular syndrome" OR "plurimetabolic syndrome"). No language and publication date restrictions were set. Additionally, a manual hand-search on Google was performed to identify studies outside the searched databases. After duplicates were removed, articles were screened based on their titles and abstracts. Studies with available full-texts were then retrieved and evaluated according to the eligibility criteria. The initial searches and overall study selection process were performed independently by two investigators (BSW and APW). Any disagreements were resolved in a consensus involving a third independent investigator (VV).

### Eligibility criteria

Research questions were structured using the Population, Index Test, Comparator, and Outcome (PICO) format (Supplementary Table 2) designed for systematic reviews of diagnostic test accuracy studies as the basis for constructing the eligibility criteria [19]. This meta-analysis included studies published in any language that: (1) used an observational design (cohort, case–control, or cross-sectional studies); (2) involved an adult population aged 18 years or older; (3) defined MetS based on any current available diagnostic criteria (e.g., Joint Interim Statement [JIS] [20], National Cholesterol Education Program-Adult Treatment Panel [NCEP-ATP] III [21], and International Diabetes Federation [IDF] [22]); and (4) investigated the diagnostic accuracy of LAP for detecting MetS in males or females. Studies were excluded if: (1) the study was a review article, case report, case series, or conference abstract; (2) the full-text was irretrievable; (3) the study involved non-general populations, including those with a specific pathologic condition (e.g., chronic kidney disease and polycystic ovary syndrome) or institutionalized residents; (4) the available data were insufficient for reconstructing the 2 × 2 diagnostic accuracy contingency table and the authors of the study did not respond after they were contacted for data request or were not willing to provide the data. In the case where studies with overlapping populations or the same characteristics were suspected (e.g., authors, population, method and period of sampling, study location, or results), the one with the largest sample size, most relevant data, and most recently published was selected, and then the rest were excluded.

### Data extraction

Two investigators (BSW and APW) performed data extraction independently based on a pre-specified checklist. Collected data were then checked for their eligibility by a third investigator (VV). The following data were obtained: the name of the first author, publication year, study location and design, characteristics of the study population, MetS diagnostic criteria, gender-specific sample size, age, values of LAP in MetS and non-MetS subjects, odds ratio (OR) between LAP and MetS, and diagnostic parameters of LAP (area under the curve [AUC], cut-off, sensitivity, and specificity). If a study used more than one diagnostic criteria for MetS, only one was selected in the following order of priority: (1) JIS; (2) NCEP-ATP III; (3) IDF; and (4) other criteria. Criteria besides JIS, NCEP-ATP III, and IDF were later classified into “others”. Given that LAP is a continuous index, only uncategorized ORs were extracted, in which LAP was treated as a continuous predictor variable during analysis. Dichotomizing or categorizing continuously distributed exposure variables has been known to cause a loss of statistical power, inaccurate estimation, and difficulty comparing results across studies due to the use of data-driven cut-points to define the categories [23, 24]. ORs adjusted for confounders were preferred over unadjusted values. To strengthen the conclusion of the current findings, the included study’s corresponding author was contacted when the 2 × 2 diagnostic contingency table of LAP in males or females could not be constructed from the reported study data. In addition, for comparative analysis purposes, all index tests used to identify MetS other than LAP with their corresponding sensitivities and specificities were extracted, but only when ≥ 4 studies reported the data of the same index tests. Eleven adiposity indicators that met this requirement were identified, including VAI, BMI, WC, WHtR, WHR, body roundness index (BRI), a body shape index (ABSI), body adiposity index (BAI), conicity index (CI), triglyceride-glucose (TyG) index, and abdominal volume index (AVI).

### Quality assessment

The quality of the included studies was assessed using the Quality Assessment of Diagnostic Accuracy Studies 2 (QUADAS-2) tool that comprises four domains: patient selection, index test, reference standard, and flow and timing [25]. Each domain is assessed in terms of risk of bias as well as concerns regarding applicability for the first three domains. The QUADAS-2 tool signaling questions for assessing the risk of bias were further tailored to have greater relevance to the current study. Additional signaling questions were formulated from studies by Bujang et al. [26], McCrea et al. [27], and Munthali et al. [28] (for full details, see Supplementary Table 3). For each signaling question, reviewers were required to respond “yes”, “no”, or “unclear.” Accordingly, the risk of bias and applicability concerns were rated as “low”, “high”, or “unclear”. A study was judged to have a low overall bias risk and concerns of applicability when all domains were rated as “low”. A high overall risk of bias was considered when the study had a high-rated risk in ≥ 1 domain or an unclear-rated risk in ≥ 3 domains, while a high concern regarding applicability was determined when the study had a high-rated concern in at least one domain. Otherwise, studies were judged as having a moderate risk or applicability concern. Quality assessments were conducted by two independent reviewers (BSW and APW). Any discordance in judgments was subsequently resolved by a third reviewer (VV).

### Statistical analysis

Statistical analyses were conducted using Review Manager ver. 5.4 (The Cochrane Collaboration, The Nordic Cochrane Centre, Copenhagen, Denmark) and STATA ver. 16.0 (Stata Corporation, College Station, TX, USA). Diagnostic test accuracy meta-analyses on LAP in identifying MetS were performed for the primary outcome. Secondary meta-analyses were additionally conducted to estimate the pooled mean difference (MD) of LAP between MetS and non-MetS subjects and the pooled OR between LAP and MetS. Males and females were analyzed separately in all outcomes. For MD meta-analysis, data that were not reported in mean and standard deviation (SD) were transformed beforehand.

Bivariate diagnostic accuracy meta-analyses were performed to obtain the pooled sensitivity, specificity, and area under the summary receiver operating characteristic (AUSROC) curve along with their corresponding 95% confidence intervals (CIs). AUSROC curve values were subsequently interpreted as similar to the AUC, where 0.5 indicates that LAP has no ability to discriminate subjects with and without MetS, 0.7 to 0.8 is considered an acceptable diagnostic power, 0.8 to 0.9 is considered excellent, and more than 0.9 is considered outstanding [29]. To assess the effect of a diagnostic threshold, the Spearman’s correlation was used by analyzing the correlation of the sensitivity and 1 – specificity between studies. The threshold effect may exist due to variations in the cut-off values ​​between studies and is considered one of the major causes of heterogeneity in diagnostic accuracy meta-analysis. A positive Spearman’s correlation coefficient with *P* < 0.05 indicates a significant threshold effect [30]. In addition to LAP, the AUSROC curves of other adiposity indicators were estimated. The *Z*-test was then adopted to compare the AUSROC curve values between LAP and these indicators [31].

Heterogeneity was assessed using the Cochran’s *Q* statistic and quantified using the Higgins’ *I*^2^ statistic as recommended by Cochrane. The *I*^2^ is a widely used measure calculated from the *Q* statistic to depict the extent of heterogeneity between studies in a meta-analysis. An *I*^2^ value of 0%, 25%, 50%, and 75% was considered as negligible, low, moderate, and high heterogeneity, respectively [32]. Since diversity of characteristics between studies was expected, a random-effects model was primarily applied for estimating the pooled effect of OR and MD meta-analyses. The random-effects model assumes that the true effect could vary between studies due to the heterogeneity among them [33]. In all analyses, a *P*-value of < 0.05 was considered statistically significant.

Publication bias in the diagnostic accuracy meta-analysis was assessed using the Deeks’ funnel plot as recommended by Cochrane [34, 35], while publication bias in the MD and OR meta-analyses was assessed visually using an inverted funnel plot and quantitatively using the Egger’s test [36]. Egger’s test has been widely used in meta-analyses and considered one of the formal statistical tests to evaluate funnel plot asymmetry [37]. Sensitivity analyses in all outcomes were carried out in four different ways by excluding: (1) each study individually (leave-one-out analysis); (2) study outliers; (3) moderate and high risk of bias studies; and (4) studies with a sample size of < 100. Subsequently, the consistency and significance of the pooled results was re-evaluated. Outliers in the diagnostic accuracy meta-analysis were identified by using a bivariate boxplot. This boxplot describes the interdependence degree between the sensitivity and specificity of each study. The inner and outer oval of the bivariate boxplot represent the median distribution and 95% CIs of all the study data points, respectively, and studies located outside the outer oval region were considered outliers. Outliers in the MD and OR meta-analyses were detected by visually examining the forest plot, where outliers were studies having their 95% CIs located outside the 95% CI of the pooled result [38].

Subgroup and meta-regression analyses were performed on the primary outcome with outliers included to search for possible causes of heterogeneity. Subgroup analyses were performed based on: (1) MetS criteria; (2) study location; (3) study design; and (4) type of population. In the case where a covariate yielded > 2 subgroups, the subgroup with the most studies included was used as the reference value. Meta-regressions were carried out for: (1) publication year; (2) mean population age; and (3) study sample size.

## Results

### Selection of studies

**Fig. 1:**
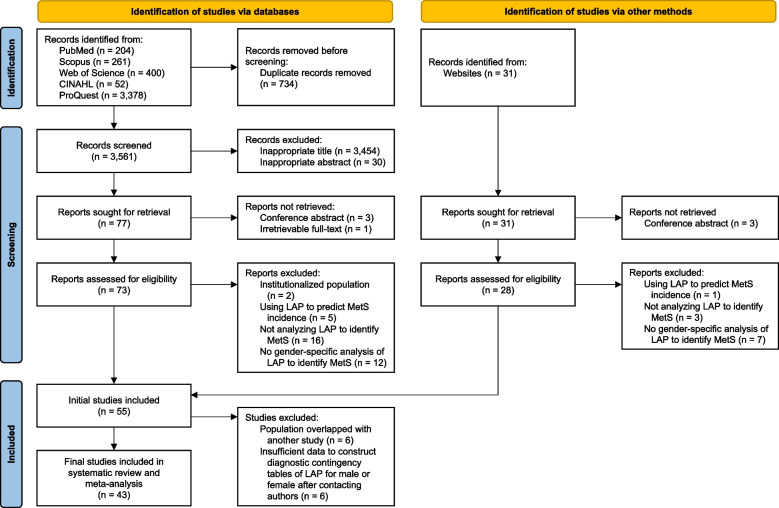
PRISMA flow diagram of the study selection process. CINAHL, Cumulative Index to Nursing and Allied Health Literature; LAP, lipid accumulation product; MetS, metabolic syndrome; PRISMA, Preferred Reporting Items for Systematic Reviews and Meta-Analyses

A PRISMA flow diagram of the overall study selection process is illustrated in Fig. 1. A total of 4,295 records were initially obtained, where 734 were then removed due to duplication. Of the remaining 3,561 articles, 3,454 and 30 were excluded respectively based on their titles and abstracts. Three conference abstracts and one article with no available full-text were not retrieved further. Afterward, the remaining 73 studies were thoroughly reviewed, and 35 were subsequently excluded due to not meeting the eligibility criteria. In addition to database searching, 31 additional records were found from websites. Of those, three conference abstracts were not retrieved, and 11 articles were excluded due to irrelevant outcomes. Fifty-five studies were initially included in this systematic review. Then, seven studies with potential overlapping populations and similar characteristics were identified. The study by Guo et al. [39] with the largest sample size was included, and the rest were excluded. Afterward, the authors of nine studies were contacted for additional data requests. Three of them responded and agreed to provide the data. Due to insufficient data to construct the diagnostic contingency table of LAP, six studies were further excluded. Ultimately, the entire screening process resulted in the inclusion of 43 eligible studies [39–81]. Of all the studies included, one by Soares et al. [73] was a gray literature.

### Characteristics, outcomes, and quality assessment of included studies

The summary characteristics of the included studies are presented in Table 1. Two studies by Ejike [50] and Tellechea et al. [77] used only male subjects, while four studies by İlhan et al. [53], Lee et al. [55], Osman et al. [67], and Shabestari et al. [70] used only female subjects. The remaining 37 studies consisted of both male and female populations. The total sample accumulated from all included studies was 202,313 adults. Of those, 98,164 were males (18,331 MetS and 79,833 non-MetS), and 104,149 were females (25,327 MetS and 78,822 non-MetS). The mean age range of the male population was 19.3 to 75.0 years, while the female population was 19.3 to 72.1 years. The study sample sizes varied from 40 to 61,283. Most studies were located in Asia (*n* = 27), followed by Africa (*n* = 8), America (*n* = 6), and Europe (*n* = 2). Only three studies used a case–control design, while the rest were cross-sectional studies (*n* = 40). All studies included diverse types of population: community residents (*n* = 19), patients attending hospitals or clinics (*n* = 18), and other populations (*n* = 6). JIS was the most commonly used diagnostic criteria for defining MetS (*n* = 19). Other criteria included NCEP-ATP III (*n* = 12), IDF (*n* = 3), and criteria included in “others” (*n* = 9). All gender-specific outcomes reported in each study, including LAP values, OR, and diagnostic accuracy parameters, are summarized in Supplementary Table 4.

**Table 1:** Characteristics and quality of the included studies

Author, Year	Study location	Study design	Population characteristics	Other adiposity indicators^a^	MetS diagnostic criteria	Gender	Sample size		Age^b^	QUADAS-2	
							**MetS**	**Non-MetS**		**Risk of bias**	**Applicability concerns**
Adejumo et al., 2019 [40]	Nigeria (Africa)	Cross-sectional	Community residents	VAI, BMI, WC, WHtR, BRI, ABSI, BAI, CI, AVI	JIS	Male	23	123	46.24 ± 14.42	Moderate	Low
						Female	100	289	47.36 ± 14.82		
Alfawaz et al., 2023 [41]	Saudi Arabia (Asia)	Cross-sectional	Community residents	VAI, BMI, WC, BRI, ABSI, BAI	NCEP-ATP III	Male	176	198	43.74 ± 12.98	Moderate	Low
						Female	232	227	40.89 ± 11.00		
Alves et al., 2021 [42]	Brazil (South America)	Cross-sectional	Outpatients attending the clinic	VAI, BMI, WC, WHtR, ABSI, CI	JIS	Male	45	34	70.9 ± 7.4	High	Moderate
						Female	71	9			
Anto et al., 2023 [43]	Ghana (Africa)	Cross-sectional	Community residents	TyG Index	IDF	Male	401	1,400	56.35 ± 8.16	Low	Low
						Female	1,481	1,458			
Banik et al., 2021 [44]	Mexico (North America)	Cross-sectional	Outpatients attending the hospital	BMI, WC	JIS	Male (age 40–59 years)	24	21	49.5 ± 4.75	High	Low
						Male (age 60–65 years)	4	11	62.5 ± 1.25		
						Female (age 20–39 years)	12	34	29.5 ± 4.75		
						Female (age 40–59 years)	60	41	49.5 ± 4.75		
						Female (age 60–65 years)	20	6	62.5 ± 1.25		
Chiang et al., 2012 [45]	Taiwan (Asia)	Cross-sectional	Patients attending the hospital	N/A	MetS criteria for Taiwanese people	Male	52	214	59.3 ± 6.9	Low	Low
						Female	53	194	58.8 ± 7.0		
Ching et al., 2020 [46]	Malaysia (Asia)	Cross-sectional	Public community	VAI, BMI, WHtR, BRI, ABSI	JIS	Male	28	68	45.97 ± 14.45	High	Low
						Female	38	139	48.39 ± 12.25		
Duan et al., 2013 [47]	China (Asia)	Cross-sectional	Community residents	N/A	JIS	Male	334	1,205	59.62 ± 12.73	Moderate	Low
						Female	242	847	57.06 ± 12.15		
Duan et al., 2021 [48]	China (Asia)	Cross-sectional	Patients attending the hospital	VAI, BMI, WC	Chinese Guidelines for the Prevention and Treatment of Type 2 Diabetes Mellitus (2017 Edition)	Male	204	293	39.20 ± 10.58	Moderate	Moderate
						Female	45	166			
Duan et al., 2022 [49]	China (Asia)	Cross-sectional	Community residents	VAI, BMI, BRI, BAI, TyG Index, AVI	Chinese Guidelines for the Prevention and Treatment of Type 2 Diabetes Mellitus (2020 Edition)	Male	171	444	58.07 ± 13.57	Low	Low
						Female	134	703	58.26 ± 13.25		
Ejike, 2011 [50]	Nigeria (Africa)	Cross-sectional	Outpatients attending the clinic	VAI, BMI, WHtR, WHR	JIS	Male	3	37	75 ± 6	High	Low
Gao et al., 2019 [51]	China (Asia)	Cross-sectional	Community residents	VAI, BMI, WC, WHtR, WHR	Chinese Guidelines for the Prevention and Treatment of Dyslipidemia in Adults (2016 Edition)	Male (Yi Nationality)	119	428	45.79 ± 14.63	Moderate	Low
						Male (Han Nationality)	182	659	46.22 ± 15.71		
						Female (Yi Nationality)	116	595	44.56 ± 14.18		
						Female (Han Nationality)	169	1,224	46.01 ± 14.49		
Gu et al., 2018 [52]	China (Asia)	Cross-sectional	Community residents	VAI, BMI, WHtR	JIS	Male	999	2,078	69.99 ± 7.35	Low	Low
						Female	1,719	1,926	70.12 ± 7.65		
Guo et al., 2016 [39]	China (Asia)	Cross-sectional	Community residents	VAI, WHtR, BAI	JIS	Male	1,078	3,311	45.93 ± 14.95	Moderate	Low
						Female	1,487	4,153	44.92 ± 13.92		
İlhan et al., 2019 [53]	Turkey (Asia)	Cross-sectional	Outpatients attending the clinic	VAI, TyG Index	NCEP-ATP III	Female	63	137	52.06 ± 5.82	High	Moderate
Jian et al., 2022 [54]	China (Asia)	Cross-sectional	Community residents	WHtR, ABSI, CI, TyG Index	JIS	Male	309	1,865	32.59 ± 14.03	Low	Low
						Female	382	1,759	31.94 ± 12.96		
Lee et al., 2018^c^ [55]	South Korea (Asia)	Cross-sectional	Patients attending the hospital	N/A	NCEP-ATP III with population-specific WC (JIS)	Female	455	3,481	52.14 ± 10.97	Moderate	Low
Li et al., 2022 [56]	United States (North America)	Cross-sectional	Community residents	VAI, TyG Index	NCEP-ATP III	Male	560	1,333	40.71 ± 22.73	Low	Low
						Female	529	1,472			
Li et al., 2023 [57]	China (Asia)	Cross-sectional	Community residents	VAI, BMI, WC, WHtR, BRI, ABSI, CI, TyG Index	NCEP-ATP III	Male	1,331	3,009	60.29 ± 9.33	Low	Low
						Female	2,629	2,488	58.41 ± 9.81		
Liu et al., 2017 [58]	China (Asia)	Case-control	Patients attending the hospital	VAI	JIS	Male	127	55	67.74 ± 7.51	High	Low
						Female	61	53			
Liu et al., 2021 [59]	China (Asia)	Cross-sectional	Patients attending the hospital	VAI	JIS	Male	5	230	19.3 ± 0.8	Moderate	Low
						Female	4	209	19.3 ± 0.8		
Llinás et al., 2017 [60]	Spain (Europe)	Cross-sectional	Company workers	VAI, BMI, WHtR, WHR, BRI, ABSI, BAI, CI, AVI	NCEP-ATP III	Male	3,195	31,847	40.06 ± 10.66	Low	Low
						Female	1,089	25,152	39.24 ± 10.30		
Luo et al., 2019 [61]	China (Asia)	Cross-sectional	Community residents	N/A	MetS Definition of Diabetes Branch of Chinese Medical Association	Male	825	1,923	60.06 ± 8.79	Moderate	Low
						Female	1,834	5,466	57.15 ± 8.45		
Mosad et al., 2023 [62]	Sudan (Africa)	Case-control	Patients attending the hospital	VAI, BMI, WC, WHtR	NCEP-ATP III	Male	77	77	54.70 ± 7.39	High	Low
						Female	133	133	53.50 ± 7.67		
Motamed et al., 2016 [63]	Iran (Asia)	Cross-sectional	Community residents	N/A	JIS	Male	1,114	2,005	44.77 ± 16.77	Moderate	Low
						Female	971	1,421	43.78 ± 15.43		
Musa et al., 2023 [64]	Nigeria (Africa)	Cross-sectional	University students	VAI, BMI	IDF	Male	11	124	25.65 ± 5.56	High	Low
						Female	14	51	24.11 ± 4.60		
Nwankwo et al., 2023^c^ [65]	Nigeria (Africa)	Cross-sectional	Outpatients attending the hospital	BMI, WHtR, BRI, ABSI, BAI, CI, AVI	IDF	Male	218	2,530	35 ± 7.5	Low	Low
						Female	476	944			
Omuse et al., 2017 [66]	Kenya (Africa)	Cross-sectional	Public community	VAI, BMI, WC	JIS	Male	64	191	38.27 ± 8.00	Low	Low
						Female	71	202	39.11 ± 8.11		
Osman et al., 2020 [67]	Sudan (Africa)	Cross-sectional	Patients attending the hospital	VAI, BMI, WC, BAI	NCEP-ATP III	Female	149	141	53.96 ± 7.5	Moderate	Low
Rabiei et al., 2021 [68]	Iran (Asia)	Cross-sectional	Community residents	VAI, WHtR, WHR, BRI, ABSI, BAI	NCEP-ATP III	Male	454	712	69.54 ± 6.44	Low	Low
						Female	849	407	69.16 ± 6.35		
Rajendran et al., 2022 [69]	India (Asia)	Cross-sectional	Patients attending the hospital	BMI, WC, WHtR, WHR, TyG Index	JIS	Male	55	95	29.9 ± 6.2	Low	Low
						Female	42	108	30.2 ± 6.3		
Shabestari et al., 2016 [70]	Iran (Asia)	Cross-sectional	Outpatients attending the clinic	N/A	NCEP-ATP III	Female	109	155	53.98 ± 5.57	High	Low
Shao et al., 2023 [71]	China (Asia)	Cross-sectional	Community residents	BMI, WC, WHtR, WHR	JIS	Male	3,886	10,505	56.61 ± 12.42	Low	Low
						Female	7,348	13,707	54.93 ± 12.40		
Shin et al., 2019 [72]	South Korea (Asia)	Cross-sectional	Patients attending the hospital	VAI, WHtR, TyG Index	NCEP-ATP III with population-specific WC (JIS)	Male	1,237	8,506	51.07 ± 9.02	Low	Low
						Female	651	5,096	51.37 ± 9.23		
Soares, 2016 [73]	Brazil (South America)	Cross-sectional	University workers	N/A	JIS	Male	39	41	41.28 ± 9.94	High	Low
						Female	42	89			
Su et al., 2020 [74]	China (Asia)	Cross-sectional	Community residents	VAI, BMI, WC, WHtR, CI	Chinese Guidelines for the Prevention and Treatment of Dyslipidemia in Adults (2016 Edition)	Male	186	356	72.12 ± 5.97	Moderate	Low
						Female	274	518	72.13 ± 6.07		
Talavera et al., 2022 [75]	Peru (South America)	Cross-sectional	Community residents	VAI, BMI, TyG Index	NCEP-ATP III	Male	107	1,829	42.30 ± 18.70	Low	Low
						Female	500	1,555	39.43 ± 14.95		
Taverna et al., 2011 [76]	Spain (Europe)	Cross-sectional	Community residents	N/A	NCEP-ATP III	Male	53	299	53.5 ± 11.7	High	Low
						Female	64	352	54.5 ± 11.5		
Tellechea et al., 2009 [77]	Argentina (South America)	Cross-sectional	Blood donors	N/A	NCEP-ATP III	Male	158	443	36.9 ± 10.8	High	Moderate
Xiang et al., 2012^c^ [78]	China (Asia)	Cross-sectional	Patients attending the hospital	N/A	MetS Definition of Diabetes Branch of Chinese Medical Association	Male	221	861	44.8 ± 13.0	High	Low
						Female	212	1,210	45.2 ± 12.9		
Yin et al., 2018 [79]	China (Asia)	Cross-sectional	Patients attending the hospital	VAI	Diagnostic Difference and Rationality Study of the MetS between the Two Diagnostic Criteria of the IDF and the NCEP III of the United States	Male	53	50	56.07 ± 15.75	High	Low
						Female	29	50			
Zhang et al., 2017 [80]	China (Asia)	Cross-sectional	Community residents	VAI, BMI, WC, WHR	JIS	Male	104	216	67.1 ± 7.5	Low	Low
						Female	196	264	63.5 ± 7.3		
Zhang et al., 2019 [81]	China (Asia)	Case-control	Patients attending the hospital	N/A	Chinese Guidelines for the Prevention and Treatment of Alcoholic Liver Disease (2018 Edition)	Male	99	207	69.99 ± 7.34	High	Moderate
						Female	172	191	70.12 ± 7.64		

^a^Only indicators used in the current meta-analyses are reported

^b^Data are presented in mean ± SD

^c^Authors that provided additional data on requests

*ABSI* A body shape index, *AVI* Abdominal volume index, *BAI* Body adiposity index, *BMI* Body mass index, *BRI* Body roundness index, *CI* Conicity index, *IDF* International Diabetes Federation, *JIS* Joint Interim Statement, *MetS* Metabolic syndrome, *N/A* Not applicable or not available, *NCEP-ATP* National Cholesterol Education Program-Adult Treatment Panel, *QUADAS-2* Quality Assessment of Diagnostic Accuracy Studies 2, *SD* Standard deviation, *TyG* Triglyceride-glucose, *VAI* Visceral adiposity index, *WC* Waist circumference, *WHR* Waist-to-hip ratio, *WHtR* Waist-to-height ratio

The overall quality of each study based on the QUADAS-2 tool is provided in Table 1, while the details of the assessment results viewed from each domain are shown in Supplementary Fig. 1 in Additional file 1. In terms of overall bias, 16 studies had a low risk, 12 had a moderate risk, and 15 had a high risk. The risk of bias was low for the patient selection domain in 30 studies, the index test domain in 19 studies, and the reference standard domain in 23 studies. The risk of bias in the flow and timing domain was high in only one study, and the rest were low. Assessments on overall concerns of applicability showed low results for 38 studies.

### Pooled MD meta-analysis

The number of studies included in the pooled MD meta-analysis for male was 18 (Supplementary Fig. 2A) with a total of 54,335 subjects (7,671 MetS and 46,664 non-MetS). The result showed that LAP in men with MetS was significantly higher than in those without MetS by 45.92 units (95% CI: 36.11–55.72; *P* < 0.001). The heterogeneity level was high (*I*^2^ = 99%). In the analysis for female, 21 studies involving 50,751 subjects (9,602 MetS and 41,149 non-MetS) were included (Supplementary Fig. 2B). LAP in women with MetS was also significantly higher than in those without MetS by 41.70 units (95% CI: 37.16–46.24; *P* < 0.001). The level of heterogeneity was high (*I*^2^ = 97%). The funnel plots for publication bias analysis were somewhat asymmetrical for male (Supplementary Fig. 3A) and female (Supplementary Fig. 3B) analyses. Nevertheless, the Egger’s tests showed insignificant results for both male (*Z*: 0.72; *P* = 0.473) and female (*Z*: 1.45; *P* = 0.147), suggesting no potential publication bias. In the male analysis, six studies [40, 50, 52, 60, 65, 75] were detected as outliers (Supplementary Fig. 2A), while in female (Supplementary Fig. 2B), eight studies [40, 52, 55, 60, 62, 65, 67, 75] were detected as outliers. Sensitivity analyses using leave one-out and other methods showed that the significance of the results for male and female was robust. The summary of the sensitivity analysis results for MD meta-analyses is shown in Supplementary Table 5.

### Pooled OR meta-analysis

Six studies with a total of 17,857 subjects (4,910 MetS and 12,947 non-MetS) were included in the male OR meta-analysis (Supplementary Fig. 4A). The result showed that LAP had a significant association with MetS in men (OR: 1.07; 95% CI: 1.06–1.09; *P* < 0.001), with a high level of heterogeneity (*I*^2^ = 83%). In the female analysis (Supplementary Fig. 4B), seven studies involving 29,018 subjects (8,835 MetS and 20,183 non-MetS) were included. LAP was also significantly associated with MetS in women (OR: 1.08; 95% CI: 1.07–1.10; *P* < 0.001). The heterogeneity level was high (*I*^2^ = 95%). Although the funnel plots (Supplementary Fig. 5A and Supplementary Fig. 5B) showed a rather asymmetrical distribution of studies, Egger’s test results showed no potential publication bias for both male (*Z*: 1.14; *P* = 0.255) and female analyses (*Z*: 1.55; *P* = 0.122). No outliers were found in male analysis (Supplementary Fig. 4A), while in female analysis, one study by Li et al. (2022) [56] was detected as an outlier (Supplementary Fig. 4B). There were no studies with a sample size of < 100 in both analyses. Results of the sensitivity analyses using leave-one-out and other methods showed no substantial change in the pooled results for male and female. Supplementary Table 6 summarizes the sensitivity analysis results for all OR meta-analyses.

### Diagnostic accuracy meta-analysis

**Fig. 2:**
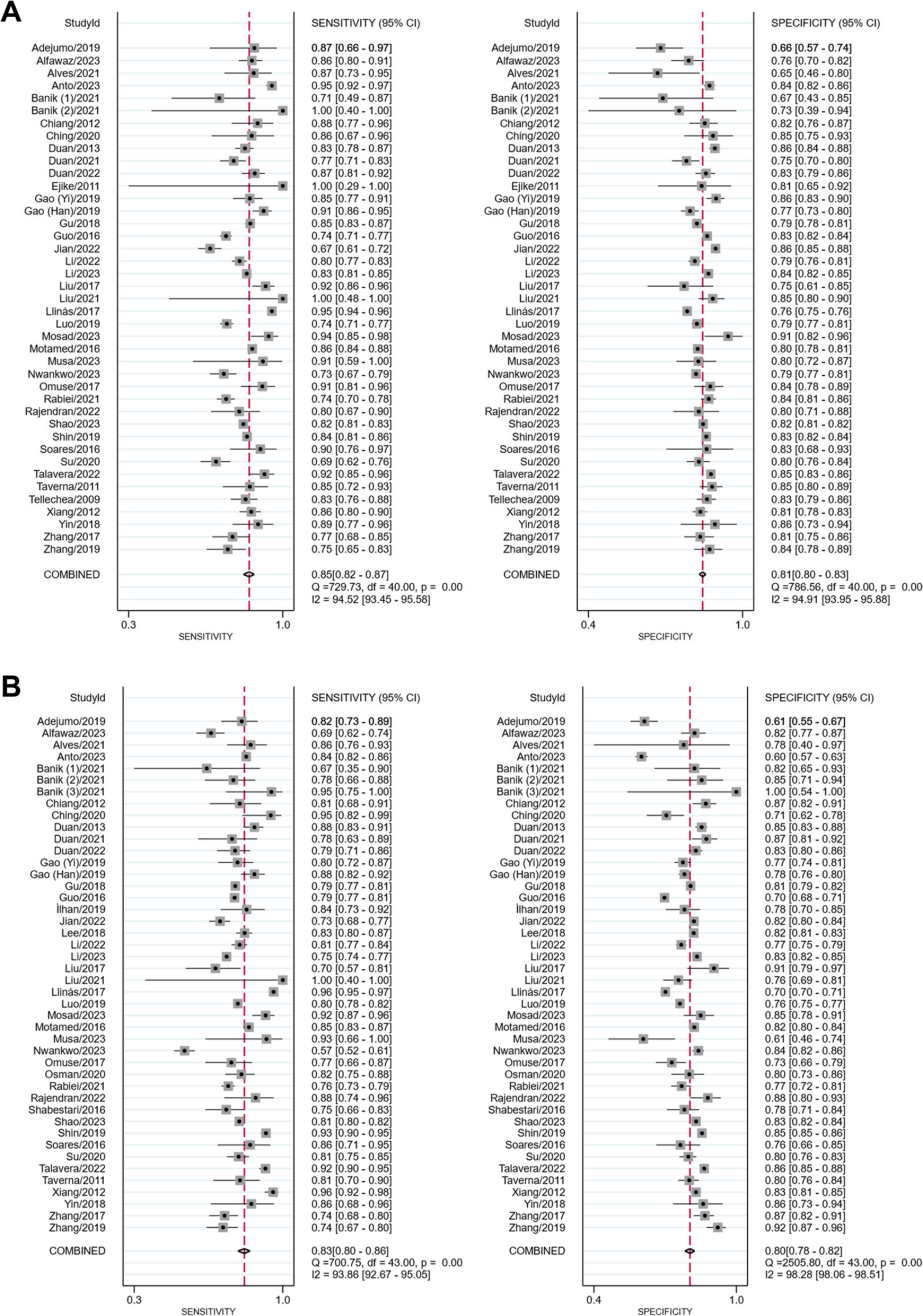
Forest plots of the pooled sensitivity and specificity of LAP for the screening of MetS in (**A**) men and (**B**) women. CI, confidence interval; LAP, lipid accumulation product; MetS, metabolic syndrome

**Fig. 3:**
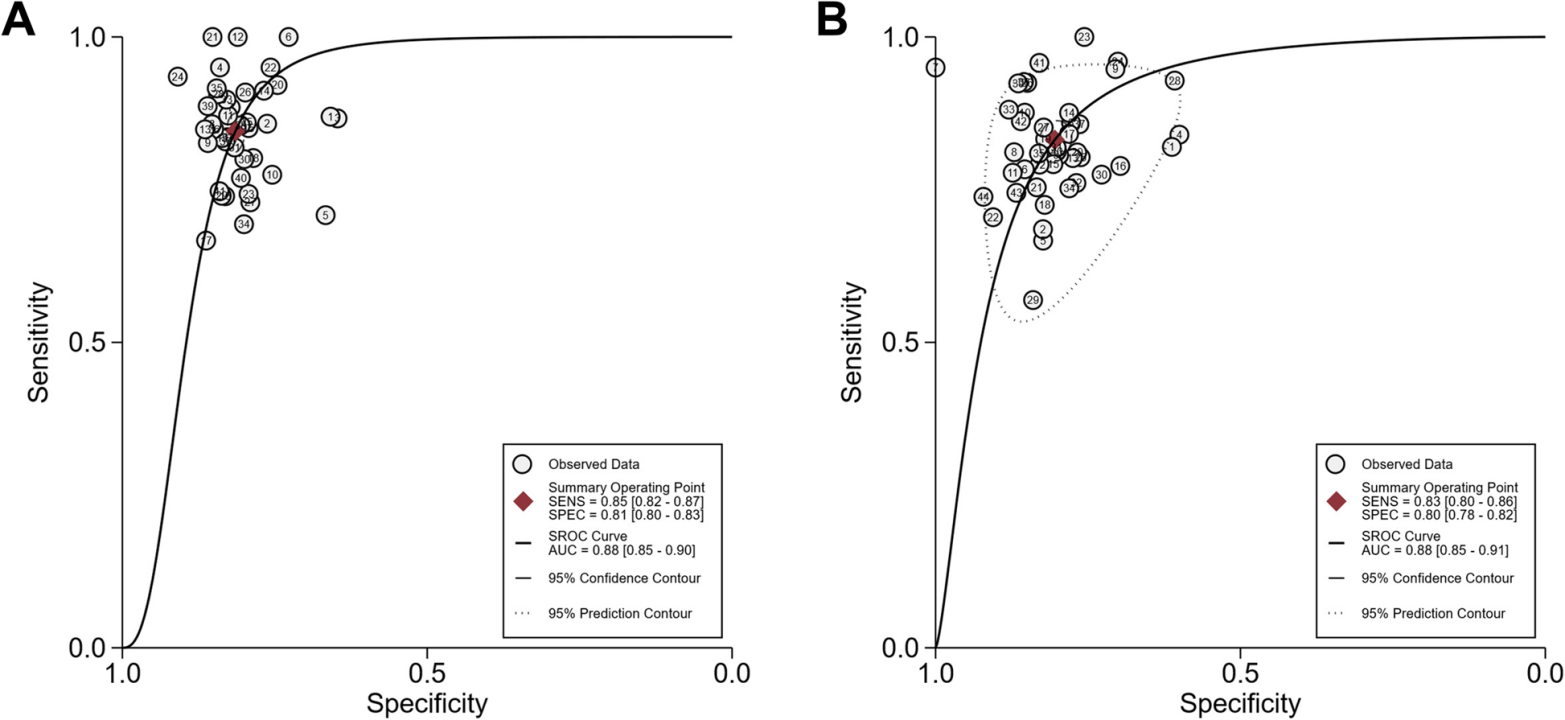
AUSROC curves of LAP for the screening of MetS in (**A**) men and (**B**) women. AUC, area under the curve; AUSROC, area under the summary receiver operating characteristic; LAP, lipid accumulation product; MetS, metabolic syndrome; SENS, sensitivity; SPEC, specificity; SROC, summary receiver operating characteristic

Thirty-nine studies involving 98,164 participants (18,331 MetS and 79,833 non-MetS) were included in the male diagnostic accuracy analysis. For detecting MetS in men, LAP had a pooled sensitivity of 85% ​​(95% CI: 82%–87%; *I*^2^ = 95%) and a pooled specificity of 81% (95% CI: 80%–83%; *I*^2^ = 95%; Fig. 2A). The diagnostic accuracy meta-analysis for female included 41 studies with 104,149 participants (25,327 MetS and 78,822 non-MetS). The pooled sensitivity and specificity of LAP in women were 83% (95% CI: 80%–86%; *I*^2^ = 94%) and 80% (95% CI: 78%–82%; *I*^2^ = 98%), respectively (Fig. 2B). The AUSROC curve analysis showed a value of 0.88 (95% CI: 0.85–0.90; Fig. 3A) for male and 0.88 (95% CI: 0.85–0.91; Fig. 3B) for female, indicating that LAP had an excellent screening accuracy for MetS in both genders. The included studies in both analyses used a varied range of LAP cut-off values, which may cause a threshold effect. Nonetheless, Spearman’s analyses showed weak and insignificant correlations for both male (*r*: 0.008; *P* = 0.961) and female (*r*: -0.163; *P* = 0.289), suggesting the heterogeneity was unlikely to be caused by a threshold effect. Results of the Deeks’ funnel plot for both male (*P* = 0.38; Supplementary Fig. 6A) and female analyses (*P* = 0.65; Supplementary Fig. 6B) indicated no publication bias.

Sensitivity analysis results for the diagnostic accuracy outcomes are summarized in Supplementary Table 7. Leave-one-out analyses showed that the pooled diagnostic accuracies in male and female were robust. The bivariate boxplot for male (Supplementary Fig. 7A) revealed six study outliers [40, 42, 44, 54, 60, 62] and the bivariate boxplot for female (Supplementary Fig. 7B) showed ten study outliers [40, 43, 44, 46, 58, 60, 64, 65, 78, 81]. Exclusion of the outliers, studies with a high risk of bias, and a sample size of < 100 showed no substantial change in the pooled results for both male and female analyses. It is worth noting that there was also no overestimation of the LAP diagnostic accuracies due to the moderate and high risk of bias studies.

### Subgroup and meta-regression analyses

Details of the subgroup and meta-regression analysis results are presented in Table 2. In male and female analyses, there were significant differences in the sensitivity and specificity of LAP between studies that used JIS and other criteria (*P* < 0.01), studies conducted in Asia and outside Asia (*P* < 0.01), and studies involving community residents and other populations (*P* < 0.01). Additionally, the subgroup analysis in female showed significant differences in the accuracy of the LAP between cross-sectional and case–control studies (*P* = 0.02). All meta-regression analyses showed that publication year, mean population age, and study sample size had no significant influence in the pooled results of both genders.

**Table 2:** Subgroup and meta-regression analyses for diagnostic accuracy meta-analyses of LAP for the screening of MetS

Subgroup and meta-regression variables	Male					Female				
	**Number of studies**	**Total sample**	**% Sn (95% CI)**	**% Sp (95% CI)**	***P*-value**	**Number of studies**	**Total sample**	**% Sn (95% CI)**	**% Sp (95% CI)**	***P*-value**
MetS Diagnostic Criteria										
JIS	18	40,075	83 (80–86)	81 (79–83)	ref	18	47,805	83 (79–86)	81 (77–83)	ref
NCEP-ATP III	9	45,858	87 (81–91)	82 (79–84)	< 0.01	11	38,565	84 (78–89)	80 (77–83)	< 0.01
IDF	3	4,684	88 (80–95)	81 (77–86)	< 0.01	3	4,424	77 (65–88)	70 (60–81)	< 0.01
Others	9	7,547	83 (78–87)	81 (79–83)	< 0.01	9	13,355	83 (78–87)	83 (79–86)	< 0.01
Study Location										
Asia	24	52,842	82 (79–84)	82 (81–83)	ref	27	67,410	82 (79–85)	82 (80–84)	ref
America	6	4,649	84 (79–88)	81 (78–84)	< 0.01	5	4,440	86 (81–90)	81 (77–84)	< 0.01
Europe	2	35,394	92 (87–97)	80 (75–84)	< 0.01	2	26,657	92 (86–98)	75 (67–83)	< 0.01
Africa	7	5,279	90 (82–94)	81 (76–85)	< 0.01	7	5,642	82 (73–89)	73 (65–80)	< 0.01
Study Design										
Cross-sectional	36	97,522	84 (82–87)	81 (80–83)	ref	38	103,406	83 (80–86)	80 (78–82)	ref
Case-control	3	642	88 (81–95)	84 (79–89)	0.42	3	743	81 (71–91)	90 (85–95)	0.02
Population Characteristics										
Community residents	19	46,310	83 (79–86)	82 (80–83)	ref	19	62,087	81 (78–83)	79 (76–82)	ref
Patients attending hospitals or clinics	14	15,645	84 (80–88)	82 (80–84)	< 0.01	17	15,175	84 (79–88)	84 (82–86)	< 0.01
Others	6	36,209	90 (84–93)	81 (77–84)	< 0.01	5	26,887	91 (87–95)	71 (63–78)	< 0.01
Meta-Regressions										
Year of publication	39	98,164	85 (82–87)	81 (80–83)	0.77	41	104,149	83 (80–86)	80 (78–82)	0.30
Mean population age	39	98,164	85 (82–87)	81 (80–83)	0.89	41	104,149	83 (80–86)	80 (78–82)	0.19
Sample size	39	98,164	85 (82–87)	81 (80–83)	0.10	41	104,149	83 (80–86)	80 (78–82)	0.10

*CI* Confidence interval, *IDF* International Diabetes Federation, *JIS* Joint Interim Statement, *LAP* Lipid accumulation product, *MetS* Metabolic syndrome, *NCEP-ATP* National Cholesterol Education Program-Adult Treatment Panel, *ref* Reference, *Sn* Sensitivity, *Sp* Specificity

### Comparison of diagnostic accuracy between LAP and other adiposity indicators for MetS

For comparative analyses, the diagnostic accuracies of indicators other than LAP reported by the included studies were pooled (Table 3). Results showed that the AUSROC curves of LAP in both men (0.88) and women (0.88) are significantly higher than most of the indicators, such as BMI, WHtR, WHR, BRI, ABSI, BAI, and CI (*P* < 0.05). The AUSROC curve of LAP was also higher than that of WC (*P* = 0.019) and AVI (*P* < 0.001) in female. Although there were no statistically significant differences, the AUSROC curve of LAP in male (0.88) and female (0.88) was yet higher than that of VAI in male (0.87; *P* = 0.880), VAI in female (0.85; *P* = 0.117), and TyG index in male (0.86; *P* = 0.378). Contrarily, the AUSROC curve of LAP in male (0.88) was lower than that of AVI (0.92; *P* = 0.039) which was obtained from analyzing only four studies.

**Table 3:** Comparison of the AUSROC curves of LAP and other adiposity indicators for the screening of MetS

Indicators	Male					Female				
	**Number of studies**	**Total sample**	**AUSROC curve (95% CI)**	***Z***	***P*-value**	**Number of studies**	**Total sample**	**AUSROC curve (95% CI)**	***Z***	***P*-value**
LAP	39	98,164	0.88 (0.85–0.90)	ref	ref	41	104,149	0.88 (0.85–0.91)	ref	ref
VAI	24	66,747	0.87 (0.84–0.90)	0.151	0.880	25	58,711	0.85 (0.81–0.88)	1.567	0.117
BMI	21	66,336	0.81 (0.77–0.84)	3.005	0.003	21	66,249	0.79 (0.75–0.82)	4.016	< 0.001
WC	12	22,636	0.87 (0.84–0.90)	0.359	0.720	13	31,646	0.83 (0.80–0.86)	2.337	0.019
WHtR	17	79,666	0.81 (0.77–0.84)	3.031	0.002	16	76,220	0.84 (0.80–0.87)	2.119	0.034
WHR	7	52,498	0.70 (0.66–0.74)	7.081	< 0.001	6	51,267	0.78 (0.74–0.82)	4.295	< 0.001
BRI	8	44,527	0.83 (0.80–0.86)	2.055	0.040	8	35,896	0.79 (0.75–0.82)	4.073	< 0.001
ABSI	9	46,165	0.69 (0.65–0.73)	7.275	< 0.001	9	37,280	0.64 (0.60–0.68)	9.298	< 0.001
BAI	7	44,480	0.72 (0.68–0.76)	6.316	< 0.001	8	36,532	0.71 (0.67–0.75)	6.834	< 0.001
CI	7	45,071	0.74 (0.70–0.78)	5.672	< 0.001	7	36,180	0.67 (0.63–0.71)	8.320	< 0.001
TyG Index	8	22,652	0.86 (0.83–0.89)	0.882	0.378	9	21,187	0.88 (0.84–0.90)	0.353	0.724
AVI	4	38,551	0.92 (0.89–0.94)	-2.069	0.039	4	28,887	0.72 (0.68–0.76)	6.508	< 0.001

*ABSI* A body shape index, *AUSROC* Area under the summary receiver operating characteristic, *AVI* Abdominal volume index, *BAI* Body adiposity index, *BMI* Body mass index, *BRI* Body roundness index, *CI* Confidence interval or conicity index, *LAP* Lipid accumulation product, *MetS* Metabolic syndrome, *ref* Reference, *TyG* Triglyceride-glucose, *VAI* Visceral adiposity index, *WC* Waist circumference, *WHR* Waist-to-hip ratio, *WHtR* Waist-to-height ratio

## Discussion

### Main findings

The current meta-analysis demonstrated significantly higher mean values of LAP in male and female patients with MetS than those without MetS. LAP was also positively associated to MetS in both men and women, indicating that an increase in LAP would increase the risk of having MetS. As a screening tool for MetS, LAP had moderate-to-high sensitivities and specificities with AUSROC curves of 0.88 in both men and women, denoting that LAP had an excellent performance in detecting MetS.

LAP was first introduced as a more effective tool than BMI in detecting cardiovascular risks in adults, which combined WC and TG to describe the anatomical and physiological changes associated with visceral fat deposition. These two measurements are used to indicate the human body’s capacity to store fat reserves. Hence, an increase in LAP may reflect excessive lipid in ectopic tissues, such as the liver, skeletal muscles, heart, blood vessels, kidneys, and pancreas, or also referred to as visceral adiposity [10]. LAP also significantly correlates to several components of MetS, including blood pressure, blood glucose, and HDL-C [12]. This evidence may explain the significant association between LAP and MetS and the high screening accuracy of LAP.

According to the subgroup analysis, the diagnostic accuracies of LAP varied based on the criteria for defining MetS. This finding may be caused by the difference in thresholds of each diagnostic criterion to define the components of MetS. In addition, there was a noticeable contrast in the indicator used for defining obesity between IDF, which uses BMI [22], and JIS and NCEP-ATP III, which use WC [20, 21]. Furthermore, the current results showed differences in the diagnostic accuracy of LAP based on the study region. This finding could be explained by the differences in races and ethnicities between the study populations. Nazare et al. [82] found a distinct pattern of visceral fat tissue distribution identified by computed tomography in various races and ethnicities. It is also to be noted that the current subgroup analysis results were in line with a previous meta-analysis investigating the screening accuracy of VAI, another adiposity indicator similar to LAP [83]. Also, the meta-regression analysis showed that age had no significant influence on the sensitivity and specificity of LAP in men and women. This finding indicates that LAP had a consistent performance across different adult age groups.

### Application of LAP for the screening of MetS

Aside from LAP, there were numerous obesity- and lipid-related indicators that have been investigated as a screening tool for MetS, such as VAI, BMI, WC, WHtR, WHR, BRI, ABSI, BAI, CI, TyG index, and AVI [40, 69]. Several of these, including BMI, WHtR, WHR, BRI, ABSI, BAI, CI, and AVI, are indicators developed only using anthropometric measurements, such as WC, weight, and height. The TyG index was derived from only biochemical measurements, that is blood glucose and TG. Recently, researchers have become more interested in combining anthropometric measurements and biochemical markers as a single indicator, which from then, LAP [10] and VAI [84] were developed.

This meta-analysis demonstrated that LAP had a superior diagnostic ability for MetS compared to BMI, WHtR, WHR, BRI, ABSI, BAI, and CI. However, the performance of LAP was not significantly different from that of VAI in men and women. A meta-analysis in 2021 by Bijari et al. [83] found that VAI had an AUSROC curve of 0.847 in detecting MetS. When qualitatively compared with this study’s findings, LAP showed a higher diagnostic performance. Despite no difference in accuracies according to this study’s findings, LAP can be considered a more practical tool than VAI to be used in daily clinical practice. LAP requires fewer factors for its derivation (i.e., WC and TG), while VAI has a more complex formula and is comprised of several factors (i.e., WC, TG, BMI, and HDL-C) [10, 84]. This evidence further supports LAP as an efficient marker, reducing the cumulative cost of MetS screening if used in a large population [50].

Besides VAI, the AUSROC curve of LAP was not significantly different from that of TyG index in men and women and was lower than that of AVI in men. The most recent meta-analysis in 2022 by Nabipoorashrafi et al. [85] found that TyG index had an AUSROC curve of 0.90 for detecting MetS in men from analyzing five studies. In women, from five studies, the study found an AUSROC curve of 0.87. When qualitatively compared, LAP had a higher diagnostic value in women and lower in men, but with a more consistent performance between both genders (0.88). Furthermore, although LAP had a lower diagnostic performance than AVI in men, the AUSROC curve of AVI in men (0.92) and women (0.72) did not demonstrate a consistent diagnostic value. These findings suggest that LAP may preferably be used than other indicators for detecting MetS universally in men and women. Additionally, LAP requires a lesser cost than the TyG index due to the involvement of only one biochemical measurement.

There was also no significant difference in the diagnostic performance of LAP and WC in men. This finding is interesting, considering that WC only requires one anthropometric measurement, making it a more cost-effective indicator. However, previous studies argued that WC is not a reliable parameter to comprehensively reflect metabolic abnormality since it cannot distinguish between subcutaneous and visceral fat tissues [11, 86]. It has been known that the most important factor in the pathogenesis of MetS is visceral lipid accumulation [87]. Hamdy et al. [88] also showed that visceral adiposity correlates more strongly to cardiometabolic risks than subcutaneous fat tissues. On the other hand, LAP was found to have a strong positive correlation to the visceral to subcutaneous fat area ratio assessed using computed tomography [89]. Computed tomography itself is considered the gold standard in measuring abdominal fat composition, including the visceral fat level [90]. Hence, LAP can be argued as a more reliable tool for identifying MetS. As previously stated, BMI, WHtR, WHR, BRI, ABSI, BAI, CI, and AVI are indicators derived from anthropometric measurements, meaning that they would require lesser costs as a screening tool. However, in terms of accuracy, LAP, with the addition of only a single biochemical measurement for its calculation, performed favorably better than these indicators, suggesting its effectiveness for the screening of MetS.

The clinical implementation of LAP as a screening tool in practice will need a particular cut-off value to distinguish patients with MetS from those without MetS. To determine this value with the best diagnostic accuracies, it would require enough studies reporting similar cut-offs, which were then pooled together in a subgroup analysis [83]. However, the current included studies reported various values as the authors used a data-driven threshold to demonstrate the best sensitivity and specificity, also known as the optimal cut-off value. The variability of cut-off values between studies might be due to differences in races, ethnicities, lifestyles, MetS diagnostic criteria, gender, and age [83]. As an example, Nascimento-Ferreira et al. [13] reported various optimal cut-offs for LAP based on gender and age groups (i.e., 64.1 for men ≤ 50 years, 36.4 for men > 50 years, 38 for women ≤ 50 years, and 34.2 for women > 50 years). Nevertheless, threshold effect analyses showed that this variability of cut-offs did not significantly affect the meta-analysis results.

### Strengths and limitations

This study is the first systematic review and meta-analysis that comprehensively investigated LAP as a potential screening tool for MetS and its comparison with other adiposity indicators. The total sample accumulated for both genders was appreciable (> 95,000), supported by the wide range of areas covered in Asia, America, Africa, and Europe, to value the utility of a screening tool in a large population. The current meta-analysis also revealed no potential publication bias and included studies with languages not limited only to English, minimizing the effect of language bias due to the selection of studies published in certain languages [91]. Yet, some limitations still exist. First, this meta-analysis showed a high heterogeneity between studies. In this regard, subgroup and meta-regression analyses have been performed to identify the potential sources of heterogeneity. The results revealed that different MetS diagnostic criteria, study regions, and population characteristics in male and female analyses significantly influenced the pooled accuracies. Additionally, the study design was significantly associated with the pooled effect in the female analysis. Second, the quality of most studies was yet low to moderate. Nevertheless, sensitivity analyses have showed that the moderate- and high-risk studies did not cause an overestimation of the pooled results. Third, although the current study has attempted to statistically compare the diagnostic performance of LAP with other adiposity indicators, the data for the other indicators was obtained limited to studies included in the current meta-analysis. Hence, serving as a pilot study, there still might be a possibility of an underestimation or overestimation of their pooled diagnostic accuracies from the true values. Yet, the pooled accuracies of several indicators, such as VAI and TyG index, were similar to that reported in meta-analyses centered on investigating each indicator by Bijari et al. [83] and Nabipoorashrafi et al. [85]. Finally, this study is still unable to compare LAP to several indicators, such as neck circumference (NC) [92], hip circumference (HC) [65], and waist-triglyceride index (WTI) [41], due to the insufficient number of studies (*n* < 4).

## Conclusion and recommendations

As a simple and inexpensive tool, LAP had a satisfactory and consistent screening accuracy for MetS in general adult males and females, outperforming other similar adiposity indicators. Given the growing prevalence of MetS and the high economic burden due to MetS, the findings of this study may support the application of LAP in a large population screening, especially in low-resource settings. However, due to the high heterogeneity in the current meta-analysis, future larger studies are recommended to focus on comparing the screening ability of LAP for MetS among different races, ethnicities, study designs, diagnostic criteria, age groups, and other specific modifying factors that could not be identified in this study, including socioeconomic factors, lifestyle, and comorbidities. Further research is also suggested to perform direct comparative analyses between the diagnostic performance of LAP and other indicators that have or have not been investigated in this study to corroborate the current findings. At the same time, there is still in need for researchers to conduct well-designed studies aiming to establish optimal cut-off values of LAP for use in daily practice. Besides, one study by Ding et al. [93] showed an interesting finding, by which the authors found that the diagnostic accuracy of LAP for MetS is increased when combined with other markers (e.g., WC and WHtR) using a logistic regression model. Based on this finding, oencourage more studies investigating the combination of LAP with other metabolic markers or possibly modifying the formula of LAP to increase its diagnostic performance.

### Supplementary Information

Additional file 1: This file contains all supplementary tables and figures of the study.

## Data Availability

All relevant data that support the findings of this study are available within the manuscript and its supplementary information files.

## Abbreviations

ABSI: A body shape index
AUC: Area under the curve
AUSROC: Area under the summary receiver operating characteristic
AVI: Abdominal volume index
BAI: Body adiposity index
BMI: Body mass index
BRI: Body roundness index
CI: Confidence interval or conicity index
CINAHL: Cumulative Index to Nursing and Allied Health Literature
HC: Hip circumference
HDL-C: High-density lipoprotein-cholesterol
IDF: International Diabetes Federation
JIS: Joint Interim Statement
LAP: Lipid accumulation product
MD: Mean difference
MetS: Metabolic syndrome
NC: Neck circumference
NCEP-ATP: National Cholesterol Education Program-Adult Treatment Panel
OR: Odds ratio
PICO: Population, Index Test, Comparator, and Outcome
PRISMA: Preferred Reporting Items for Systematic Reviews and Meta-Analyses
PROSPERO: International Prospective Register of Systematic Reviews
QUADAS-2: Quality Assessment of Diagnostic Accuracy Studies 2
SD: Standard deviation
TG: Triglyceride
TyG: Triglyceride-glucose
VAI: Visceral adiposity index
WC: Waist circumference
WHR: Waist-to-hip ratio
WHtR: Waist-to-height ratio
WTI: Waist-triglyceride index

## References

[1] Magliano DJ, Shaw JE, Zimmet PZ (2006). How to best define the metabolic syndrome. Ann Med.

[2] Misra A, Khurana L (2008). Obesity and the metabolic syndrome in developing countries. J Clin Endocrinol Metab.

[3] Scholze J, Alegria E, Ferri C, Langham S, Stevens W, Jeffries D (2010). Epidemiological and economic burden of metabolic syndrome and its consequences in patients with hypertension in Germany, Spain and Italy; a prevalence-based model. BMC Public Health.

[4] Noubiap JJ, Nansseu JR, Lontchi-Yimagou E, Nkeck JR, Nyaga UF, Ngouo AT (2022). Geographic distribution of metabolic syndrome and its components in the general adult population: A meta-analysis of global data from 28 million individuals. Diabetes Res Clin Pract.

[5] Liang X, Or B, Tsoi MF, Cheung CL, Cheung BMY (2023). Prevalence of metabolic syndrome in the United States National Health and Nutrition Examination Survey 2011–18. Postgrad Med J.

[6] Mottillo S, Filion KB, Genest J, Joseph L, Pilote L, Poirier P (2010). The metabolic syndrome and cardiovascular risk: A systematic review and meta-analysis. J Am Coll Cardiol.

[7] Gallagher EJ, LeRoith D, Karnieli E (2011). The Metabolic Syndrome-from Insulin Resistance to Obesity and Diabetes. Med Clin North Am.

[8] Tomic D, Shaw JE, Magliano DJ (2022). The burden and risks of emerging complications of diabetes mellitus. Nat Rev Endocrinol.

[9] Ngoc HN, Kriengsinyos W, Rojroongwasinkul N, Aekplakorn W (2021). Prevalence of metabolic syndrome and its prediction by simple adiposity indices in thai adults. J Heal Sci Med Res.

[10] Kahn HS (2005). The “lipid accumulation product” performs better than the body mass index for recognizing cardiovascular risk: A population-based comparison. BMC Cardiovasc Disord.

[11] Xia C, Li R, Zhang S, Gong L, Ren W, Wang Z (2012). Lipid accumulation product is a powerful index for recognizing insulin resistance in non-diabetic individuals. Eur J Clin Nutr.

[12] Vieira BA, Sauer P, Marcadenti A, Piovesan CH (2015). Association between LAP Index (Lipid Accumulation Product) and metabolic profile in hospitalized patients. Nutr Hosp.

[13] Nascimento-Ferreira MV, Rendo-Urteaga T, Vilanova-Campelo RC, Carvalho HB, da Paz OG, Paes Landim MB (2017). The lipid accumulation product is a powerful tool to predict metabolic syndrome in undiagnosed Brazilian adults. Clin Nutr.

[14] Li R, Li Q, Cui M, Yin Z, Li L, Zhong T (2018). Clinical surrogate markers for predicting metabolic syndrome in middle-aged and elderly Chinese. J Diabetes Investig.

[15] Cheng Y-L, Wang Y-J, Lan K-H, Huo T-I, Huang Y-H, Su C-W (2017). Fatty Liver Index and Lipid Accumulation Product Can Predict Metabolic Syndrome in Subjects without Fatty Liver Disease. Gastroenterol Res Pract.

[16] Endukuru CK, Gaur GS, Dhanalakshmi Y, Sahoo J, Vairappan B (2022). Cut-off values and clinical efficacy of body roundness index and other novel anthropometric indices in identifying metabolic syndrome and its components among Southern-Indian adults. Diabetol Int.

[17] Page MJ, McKenzie JE, Bossuyt PM, Boutron I, Hoffmann TC, Mulrow CD, The PRISMA (2020). statement: An updated guideline for reporting systematic reviews. BMJ.

[18] Deeks JJ, Bossuyt PM, Leeflang MM, Takwoingi Y, editors. Cochrane Handbook for Systematic Reviews of Diagnostic Test Accuracy Version 2.0. Cochrane. 2023. https://training.cochrane.org/handbook-diagnostic-test-accuracy/current. Accessed 20 Jan 2024. 10.1002/14651858.ED000163 PMC10408284 37470764

[19] Kim KW, Lee J, Choi SH, Huh J, Park SH (2015). Systematic review and meta-analysis of studies evaluating diagnostic test accuracy: a practical review for clinical researchers–part I. general guidance and tips. Korean J Radiol.

[20] Alberti KGMM, Eckel RH, Grundy SM, Zimmet PZ, Cleeman JI, Donato KA (2009). Harmonizing the metabolic syndrome: A joint interim statement of the international diabetes federation task force on epidemiology and prevention; National heart, lung, and blood institute; American heart association; World heart federation. Int Circulation.

[21] Expert Panel on Detection, Evaluation, and Treatment of High Blood Cholesterol in Adults. Executive Summary of The Third Report of The National Cholesterol Education Program (NCEP) Expert Panel on Detection, Evaluation, And Treatment of High Blood Cholesterol In Adults (Adult Treatment Panel III). J Am Med Assoc. 2001;285:2486–97. 10.1001/jama.285.19.2486 11368702

[22] Alberti KGMM, Zimmet P, Shaw J (2005). The metabolic syndrome - A new worldwide definition. Lancet.

[23] Naggara O, Raymond J, Guilbert F, Roy D, Weill A, Altman DG (2011). Analysis by categorizing or dichotomizing continuous variables is inadvisable: an example from the natural history of unruptured aneurysms. AJNR Am J Neuroradiol.

[24] Bennette C, Vickers A (2012). Against quantiles: categorization of continuous variables in epidemiologic research, and its discontents. BMC Med Res Methodol.

[25] Whiting PF, Rutjes AWS, Westwood ME, Mallett S, Deeks JJ, Reitsma JB (2011). QUADAS-2: A Revised Tool for the Quality Assessment of Diagnostic Accuracy Studies. Ann Intern Med.

[26] Bujang MA, Adnan TH (2016). Requirements for Minimum Sample Size for Sensitivity and Specificity Analysis. J Clin Diagn Res.

[27] McCrea M, Meier T, Huber D, Ptito A, Bigler E, Debert CT (2017). Role of advanced neuroimaging, fluid biomarkers and genetic testing in the assessment of sport-related concussion: a systematic review. Br J Sports Med.

[28] Munthali C, Taegtmeyer M, Garner PG, Lalloo DG, Squire SB, Corbett EL (2014). Diagnostic accuracy of the WHO clinical staging system for defining eligibility for ART in sub-Saharan Africa: a systematic review and meta-analysis. J Int AIDS Soc.

[29] Mandrekar JN (2010). Receiver operating characteristic curve in diagnostic test assessment. J Thorac Oncol.

[30] Jia M-M, Deng J, Cheng X, Yan Z, Li Q-C, Xing Y-Y (2017). Diagnostic accuracy of urine HE4 in patients with ovarian cancer: a meta-analysis. Med (United States).

[31] Feng S, Wang Z, Zhao Y, Tao C (2020). Wisteria floribunda agglutinin-positive Mac-2-binding protein as a diagnostic biomarker in liver cirrhosis: an updated meta-analysis. Sci Rep.

[32] Higgins JPT, Thompson SG, Deeks JJ, Altman DG (2003). Measuring inconsistency in meta-analyses. BMJ.

[33] Dettori JR, Norvell DC, Chapman JR (2022). Fixed-Effect vs Random-Effects Models for Meta-Analysis: 3 Points to Consider. Glob Spine J.

[34] Deeks JJ, Macaskill P, Irwig L (2005). The performance of tests of publication bias and other sample size effects in systematic reviews of diagnostic test accuracy was assessed. J Clin Epidemiol.

[35] Macaskill P, Gatsonis C, Deeks J, Harbord R, Takwoingi Y, Deeks JJ, Bossuyt PM, Gatsonis C (2010). Chapter 10: analysing and presenting results. Cochrane Handb Syst Rev Diagnostic Test Accuracy Version 10.

[36] Egger M, Smith GD, Schneider M, Minder C (1997). Bias in meta-analysis detected by a simple, graphical test. Br Med J.

[37] Simmonds M (2015). Quantifying the risk of error when interpreting funnel plots. Syst Rev.

[38] Cuijpers P. Meta-analyses in mental health research: A practical guide. Amsterdam: Vrije Universiteit Amsterdam; 2016. https://www.researchgate.net/publication/301815425_Metaanalyses_in_mental_health_research_A_practical_guide.

[39] Guo SX, Zhang XH, Zhang JY, He J, Yan YZ, Ma JL (2016). Visceral Adiposity and Anthropometric Indicators as Screening Tools of Metabolic Syndrome among Low Income Rural Adults in Xinjiang. Sci Rep.

[40] Adejumo EN, Adejumo AO, Azenabor A, Ekun AO, Enitan SS, Adebola OK (2019). Anthropometric parameter that best predict metabolic syndrome in South west Nigeria. Diabetes Metab Syndr Clin Res Rev.

[41] Alfawaz HA, Khan N, Ansari MGA, Khattak MNK, Saadawy GM, Al-Daghri NM (2023). Sex-specific cut-offs of seven adiposity indicators and their performance in predicting metabolic syndrome in Arab adults. J Clin Med.

[42] Alves LF, Cruz JO, da Costa Souza AL, de Oliveira CC (2021). Performance of adiposity indicators in predicting metabolic syndrome in older adults. Arch Endocrinol Metab.

[43] Anto EO, Frimpong J, Boadu WIO, Korsah EE, Tamakloe VCKT, Ansah E (2023). Cardiometabolic syndrome among general adult population in Ghana: The role of lipid accumulation product, waist circumference-triglyceride index, and triglyceride-glucose index as surrogate indicators. Heal Sci reports.

[44] Banik SD, Pacheco-Pantoja E, Lugo R, Gómez-De-regil L, Aké RC, González RMM (2021). Evaluation of anthropometric indices and lipid parameters to predict metabolic syndrome among adults in Mexico. Diabetes, Metab Syndr Obes Targets Ther.

[45] Chiang JK, Koo M (2012). Lipid accumulation product: a simple and accurate index for predicting metabolic syndrome in Taiwanese people aged 50 and over. BMC Cardiovasc Disord.

[46] Ching YK, Chin YS, Appukutty M, Gan WY, Chan YM (2020). Comparisons of conventional and novel anthropometric obesity indices to predict metabolic syndrome among vegetarians in Malaysia. Sci Rep.

[47] Duan FY, Li R, Zhang SH, Ren W, Wang ZH, Gong LL (2013). Lipid accumulation product as an effective marker for metabolic syndrome. Chinese J Pract Intern Med.

[48] Duan SJ, Liu ZJ, Chen JL, Yao SK (2021). Predictive Value of Lipid Accumulation Product and Visceral Fat Index for Adult Metabolic Syndrome. Chinese Gen Pract.

[49] Duan Y, Zhang W, Li Z, Niu Y, Chen Y, Liu X (2022). Predictive ability of obesity- and lipid-related indicators for metabolic syndrome in relatively healthy Chinese adults. Front Endocrinol (Lausanne).

[50] Ejike CECC (2011). Lipid Accumulation Product and Waist-To-Height Ratio Are Predictors of the Metabolic Syndrome in a Nigerian Male Geriatric Population. J Rural Trop Public Heal.

[51] Gao Y-Y, Feng X-B, Cheng Y-F, Gao Y, Tian H-M, Ren Y (2019). Predictive Value of Obesity Indicators for Metabolic Syndrome in Adults of Han and Yi Nationalities in Sichuan. J Sichuan Univ Med Sci Ed.

[52] Gu Z, Zhu P, Wang Q, He H, Xu J, Zhang L (2018). Obesity and lipid-related parameters for predicting metabolic syndrome in Chinese elderly population. Lipids Health Dis.

[53] İlhan GA, Yıldızhan B (2019). Visceral adiposity indicators as predictors of metabolic syndrome in postmenopausal women. Turkish J Obstet Gynecol.

[54] Jian L-Y, Guo S-X, Ma R-L, He J, Rui D-S, Ding Y-S (2022). Comparison of obesity-related indicators for identifying metabolic syndrome among normal-weight adults in rural Xinjiang. China BMC Public Health.

[55] Lee HJ, Jo HN, Kim YH, Kim SC, Joo JK, Lee KS (2018). Predictive value of lipid accumulation product, fatty liver index, visceral adiposity index for metabolic syndrome according to menopausal status. Metab Syndr Relat Disord.

[56] Li Y, Zheng R, Li S, Cai R, Ni F, Zheng H (2022). Association Between Four Anthropometric Indexes and Metabolic Syndrome in US Adults. Front Endocrinol (Lausanne).

[57] Li Y, Gui J, Liu H, Guo L-L, Li J, Lei Y (2023). Predicting metabolic syndrome by obesity- and lipid-related indices in mid-aged and elderly Chinese: a population-based cross-sectional study. Front Endocrinol (Lausanne).

[58] Liu C, Zhang X, Tang F, Zhang L (2017). Study on the value of lipid accumulation product and visceral adiposity index in the evaluation of metabolic syndrome in the elderly. Chinese J Difficult Complicat Cases.

[59] Liu X, Ma C, Yin F, Wang R, Lu Q, Lu N (2021). Performance of Two Novel Obesity Indicators for the Management of Metabolic Syndrome in Young Adults. Front Endocrinol (Lausanne).

[60] Llinás MG, Janer PE, Agudo SG, Casquero RG, González IC (2017). Usefulness in nursing of different anthropometric and analytical indices to assess the existence of metabolic syndrome with the NCEP ATP III and IDF criteria in Spanish Mediterranean population. Med Balear.

[61] Luo L, Niu M, Zhang N, Gao Z (2019). Value of lipid accumulation product in screening of metabolic syndrome in middle-aged and elderly people. Clin Focus.

[62] Mosad AS, Elfadil GA, Elhassan SH, Elbashir ZA, Karar T, S A Husain NEO (2023). Diagnostic performance using obesity and lipid-related indices and atherogenic index of plasma to predict metabolic syndrome in the adult sudanese population. Niger J Clin Pract.

[63] Motamed N, Razmjou S, Hemmasi G, Maadi M, Zamani F (2016). Lipid accumulation product and metabolic syndrome: A population-based study in northern Iran. Amol J Endocrinol Invest.

[64] Musa AH, Ijagila IN, Dungus MM (2023). Evaluation of Metabolic Syndrome Using Lipid Accumulation Products, Visceral Adiposity Index and Body Mass Index in Apparently Healthy Students of University of Maiduguri. East African Sch J Med Sci.

[65] Nwankwo M, Okamkpa JC, Danborno B, Opoola FO (2023). Anthropometric cut-offs for screening metabolic syndrome in a Nigerian population in southeast Nigeria. J Cardiovasc Dis Res.

[66] Omuse G, Maina D, Hoffman M, Mwangi J, Wambua C, Kagotho E (2017). Metabolic syndrome and its predictors in an urban population in Kenya: A cross sectional study. BMC Endocr Disord.

[67] Osman A, Dafalla S (2020). Adiposity indices as predictors for metabolic syndrome in postmenopausal women. Natl J Physiol Pharm Pharmacol.

[68] Rabiei N, Heshmat R, Gharibzadeh S, Ostovar A, Maleki V, Sadeghian M (2021). Comparison of anthro-metabolic indicators for predicting the risk of metabolic syndrome in the elderly population: Bushehr Elderly Health (BEH) program. J Diabetes Metab Disord.

[69] Rajendran S, Kizhakkayil Padikkal AK, Mishra S, Madhavanpillai M (2022). Association of Lipid Accumulation Product and Triglyceride-Glucose Index with Metabolic Syndrome in Young Adults: A Cross-sectional Study. Int J Endocrinol Metab.

[70] Shabestari AN, Asadi M, Jouyandeh Z, Qorbani M, Kelishadi R (2016). Association of lipid accumulation product with cardio-metabolic risk factors in postmenopausal women. Acta Med Iran.

[71] Shao Q, Li J, Wu Y, Liu X, Wang N, Jiang Y (2023). Enhanced Predictive value of lipid accumulation product for identifying metabolic syndrome in the general population of China. Nutrients.

[72] Shin KA, Kim YJ (2019). Usefulness of surrogate markers of body fat distribution for predicting metabolic syndrome in middle-aged and older Korean populations. Diabetes, Metab Syndr Obes Targets Ther.

[73] Soares LM. Produto de acumulação lipídica : acurácia para identificação de portadores da síndrome metabólica em adultos. Dissertation, Brazil, Universidade Federal de Minas Gerais. 2016. https://repositorio.ufmg.br/handle/1843/BUBD-AM7NP8. Accessed 20 Jan 2024.

[74] Su ZZ, Li B, Lyu C, Li BB, Wu YY, Wang PX (2020). Predictive value of obesity index for metabolic syndrome in elderly residents in Henan Province. Occup Heal.

[75] Talavera JE, Torres-Malca JR (2022). Diagnostic performance of lipid accumulation indices and triglyceride and glucose index for metabolic syndrome in a sample of Peruvian adult population. Rev la Fac Med Humana.

[76] Taverna MJ, Martínez-Larrad MT, Frechtel GD, Serrano-Ríos M (2011). Lipid accumulation product: A powerful marker of metabolic syndrome in healthy population. Eur J Endocrinol.

[77] Tellechea ML, Aranguren F, Martínez-Larrad MT, Serrano-Ríos M, Taverna MJ, Frechtel GD (2009). Ability of lipid accumulation product to identify metabolic syndrome in healthy men from Buenos Aires. Diabetes Care.

[78] Xiang SK, Hua F, Ren JR, Tang Y, Jiang XH (2012). Diagnostic value of lipid accumulation product in metabolic syndrome in adults. New Med.

[79] Yin L, Fan H, Dong Q, Yu J (2018). Application of LAP and VAI in the diagnosis of adult metabolic syndrome. Chinese J Conval Med.

[80] Zhang Q, Chen X, Shi D, Wang S (2017). Correlation between Different Obesity Measurement Indexes and Risk of Metabolic Syndrome in Middle and Old Aged People in Chengdu. Sichuan Med J.

[81] Zhang N, Wu F, Sun B, Liu XY, Zheng GL (2019). Characteristics of Obesity and Lipid Metabolism-related Parameters in the Patients with Metabolic Syndrome and Their Diagnostic Value. Prog Mod Biomed.

[82] Nazare J-A, Smith JD, Borel A-L, Haffner SM, Balkau B, Ross R (2012). Ethnic influences on the relations between abdominal subcutaneous and visceral adiposity, liver fat, and cardiometabolic risk profile: the International Study of Prediction of Intra-Abdominal Adiposity and Its Relationship With Cardiometabolic Risk/Intra-. Am J Clin Nutr.

[83] Bijari M, Jangjoo S, Emami N, Raji S, Mottaghi M, Moallem R (2021). The Accuracy of Visceral Adiposity Index for the Screening of Metabolic Syndrome: A Systematic Review and Meta-Analysis. Int J Endocrinol.

[84] Amato MC, Giordano C, Galia M, Criscimanna A, Vitabile S, Midiri M (2010). Visceral adiposity index: A reliable indicator of visceral fat function associated with cardiometabolic risk. Diabetes Care.

[85] Nabipoorashrafi SA, Seyedi SA, Rabizadeh S, Ebrahimi M, Ranjbar SA, Reyhan SK (2022). The accuracy of triglyceride-glucose (TyG) index for the screening of metabolic syndrome in adults: A systematic review and meta-analysis. Nutr Metab Cardiovasc Dis.

[86] Després J-P, Lemieux I, Bergeron J, Pibarot P, Mathieu P, Larose E (2008). Abdominal obesity and the metabolic syndrome: contribution to global cardiometabolic risk. Arterioscler Thromb Vasc Biol.

[87] Rochlani Y, Pothineni NV, Kovelamudi S, Mehta JL (2017). Metabolic syndrome: Pathophysiology, management, and modulation by natural compounds. Ther Adv Cardiovasc Dis.

[88] Hamdy O, Porramatikul S, Al-Ozairi E (2006). Metabolic obesity: the paradox between visceral and subcutaneous fat. Curr Diabetes Rev.

[89] Kwon S, Han AL (2019). The Correlation between the Ratio of Visceral Fat Area to Subcutaneous Fat Area on Computed Tomography and Lipid Accumulation Product as Indexes of Cardiovascular Risk. J Obes Metab Syndr.

[90] Fourman LT, Kileel EM, Hubbard J, Holmes T, Anderson EJ, Looby SE (2019). Comparison of visceral fat measurement by dual-energy X-ray absorptiometry to computed tomography in HIV and non-HIV. Nutr Diabetes.

[91] Pieper D, Puljak L (2021). Language restrictions in systematic reviews should not be imposed in the search strategy but in the eligibility criteria if necessary. J Clin Epidemiol.

[92] Kim K-Y, Moon H-R, Yun J-M (2021). Neck circumference as a predictor of metabolic syndrome in Koreans: a cross-sectional study. Nutrients.

[93] Ding YS, Li Y, Zhang XH, Ma RL, Guo H, Ma L (2021). The improved lipid accumulation product is an accurate index for predicting metabolic syndrome in the Xinjiang population. Biomed Environ Sci.

