# Supplementary material for: Gender-specific accuracy of lipid accumulation product index for the screening of metabolic syndrome in general adults: a meta-analysis and comparative analysis with other adiposity indicators
Source: Lipids Health Dis. 2024 Jun 26;23:198. doi: 10.1186/s12944-024-02190-1 (PMC11201307; doi:10.1186/s12944-024-02190-1)
Supplement: Supplementary file 1 — Additional file 1: This file contains all supplementary tables and figures of the study. [file 12944_2024_2190_MOESM1_ESM.pdf]

SUPPLEMENTARY INFORMATION

Gender-specific accuracy of lipid accumulation product index for the screening of metabolic syndrome in general adults: A meta-analysis and comparative analysis with other adiposity indicators

Bendix Samarta Witarto[1], Andro Pramana Witarto[1], Visuddho Visuddho[1], Citrawati Dyah Kencono Wungu[2,3,*], Ummi Maimunah[4], Purwo Sri Rejeki[5], Delvac Oceandy[6]

[1] Medical Program, Faculty of Medicine, Universitas Airlangga, Surabaya, Indonesia
[2] Biochemistry Division, Department of Medical Physiology and Biochemistry, Faculty of Medicine, Universitas Airlangga, Surabaya, Indonesia
[3] Institute of Tropical Disease, Universitas Airlangga, Surabaya, Indonesia
[4] Division of Gastroenterology and Hepatology, Department of Internal Medicine, Dr. Soetomo General Hospital, Faculty of Medicine, Universitas Airlangga, Surabaya, Indonesia
[5] Physiology Division, Department of Medical Physiology and Biochemistry, Faculty of Medicine, Universitas Airlangga, Surabaya, Indonesia
[6] Division of Cardiovascular Science, Manchester Academic Health Science Centre, University of Manchester, Manchester M13 9PG, UK

* **Corresponding author**
Citrawati Dyah Kencono Wungu, Biochemistry Division, Department of Medical Physiology and Biochemistry, Faculty of Medicine, Universitas Airlangga, Jl. Mayjen Prof. Dr. Moestopo 47, Surabaya, East Java 60132, Indonesia. Email: citrawati.dyah@fk.unair.ac.id

TABLE OF CONTENTS

Supplementary Material	Page
**Title, Authors, and Affiliations Page**	1
**Table of Contents**	2
**Supplementary Table 1.** PRISMA 2020 Checklist.	3
**Supplementary Table 2.** PICO framework.	7
**Supplementary Table 3.** Modified QUADAS-2 tool signaling questions for risk of bias.	8
**Supplementary Table 4.** Outcomes of included studies.	10
**Supplementary Table 5.** Sensitivity analyses for MD meta-analyses of LAP in MetS and non-MetS subjects.	16
**Supplementary Table 6.** Sensitivity analyses for OR meta-analyses between LAP and MetS.	17
**Supplementary Table 7.** Sensitivity analyses for diagnostic accuracy meta-analyses of LAP as a screening tool for MetS.	18
**Supplementary Fig. 1.** Domain-specific quality assessment results of included studies using the QUADAS-2 tool.	19
**Supplementary Fig. 2.** Forest plots of MD meta-analyses of LAP in (**A**) men and (**B**) women with and without MetS.	20
**Supplementary Fig. 3.** Funnel plots of MD meta-analyses of LAP in (**A**) men and (**B**) women with and without MetS.	21
**Supplementary Fig. 4.** Forest plots of OR meta-analyses between LAP and MetS in (**A**) men and (**B**) women.	22
**Supplementary Fig. 5.** Funnel plots of OR meta-analyses between LAP and MetS in (**A**) men and (**B**) women.	23
**Supplementary Fig. 6.** Deeks' funnel plots of diagnostic accuracy meta-analyses of LAP for detecting MetS in (**A**) men and (**B**) women.	24
**Supplementary Fig. 7.** Bivariate boxplots of diagnostic accuracy meta-analyses of LAP for detecting MetS in (**A**) men and (**B**) women.	25
**References**	26

Supplementary Table 1. PRISMA 2020 Checklist [1].

Section and Topic	Item #	Checklist item	Location where item is reported[a]
**TITLE**			
Title	1	Identify the report as a systematic review.	Page 1
**ABSTRACT**			
Abstract	2	See the PRISMA 2020 for Abstracts checklist.	Page 3
**INTRODUCTION**			
Rationale	3	Describe the rationale for the review in the context of existing knowledge.	Page 4–5
Objectives	4	Provide an explicit statement of the objective(s) or question(s) the review addresses.	Page 5
**METHODS**			
Eligibility criteria	5	Specify the inclusion and exclusion criteria for the review and how studies were grouped for the syntheses.	Page 6–7
Information sources	6	Specify all databases, registers, websites, organisations, reference lists and other sources searched or consulted to identify studies. Specify the date when each source was last searched or consulted.	Page 6
Search strategy	7	Present the full search strategies for all databases, registers and websites, including any filters and limits used.	Page 6
Selection process	8	Specify the methods used to decide whether a study met the inclusion criteria of the review, including how many reviewers screened each record and each report retrieved, whether they worked independently, and if applicable, details of automation tools used in the process.	Page 6 & Suppl. Table 2
Data collection process	9	Specify the methods used to collect data from reports, including how many reviewers collected data from each report, whether they worked independently, any processes for obtaining or confirming data from study investigators, and if applicable, details of automation tools used in the process.	Page 7
Data items	10a	List and define all outcomes for which data were sought. Specify whether all results that were compatible with each outcome domain in each study were sought (e.g. for all measures, time points, analyses), and if not, the methods used to decide which results to collect.	Page 7–8
	10b	List and define all other variables for which data were sought (e.g. participant and intervention characteristics, funding sources). Describe any assumptions made about any missing or unclear information.	Page 7–8
Study risk of bias assessment	11	Specify the methods used to assess risk of bias in the included studies, including details of the tool(s) used, how many reviewers assessed each study and whether they worked independently, and if applicable, details of automation tools used in the process.	Page 8–9 & Suppl. Table 3
Effect measures	12	Specify for each outcome the effect measure(s) (e.g. risk ratio, mean difference) used in the synthesis or presentation of results.	Page 9–10
Synthesis	13a	Describe the processes used to decide which studies were eligible for each synthesis (e.g. tabulating the study intervention	Page 7–

Section and Topic	Item #	Checklist item	Location where item is reported[a]
methods		characteristics and comparing against the planned groups for each synthesis (item #5)).	11
	13b	Describe any methods required to prepare the data for presentation or synthesis, such as handling of missing summary statistics, or data conversions.	Page 7 & 9–10
	13c	Describe any methods used to tabulate or visually display results of individual studies and syntheses.	Page 9–11
	13d	Describe any methods used to synthesize results and provide a rationale for the choice(s). If meta-analysis was performed, describe the model(s), method(s) to identify the presence and extent of statistical heterogeneity, and software package(s) used.	Page 9–10
	13e	Describe any methods used to explore possible causes of heterogeneity among study results (e.g. subgroup analysis, meta-regression).	Page 11
	13f	Describe any sensitivity analyses conducted to assess robustness of the synthesized results.	Page 10–11
Reporting bias assessment	14	Describe any methods used to assess risk of bias due to missing results in a synthesis (arising from reporting biases).	N/A
Certainty assessment	15	Describe any methods used to assess certainty (or confidence) in the body of evidence for an outcome.	N/A
**RESULTS**			
Study selection	16a	Describe the results of the search and selection process, from the number of records identified in the search to the number of studies included in the review, ideally using a flow diagram.	Page 11–12 & Fig. 1
	16b	Cite studies that might appear to meet the inclusion criteria, but which were excluded, and explain why they were excluded.	Page 11–12
Study characteristics	17	Cite each included study and present its characteristics.	Page 12–13 & Table 1
Risk of bias in studies	18	Present assessments of risk of bias for each included study.	Page 13, Table 1, & Suppl. Fig. 1
Results of individual studies	19	For all outcomes, present, for each study: (a) summary statistics for each group (where appropriate) and (b) an effect estimate and its precision (e.g. confidence/credible interval), ideally using structured tables or plots.	Suppl. Table 4
Results of	20a	For each synthesis, briefly summarise the characteristics and risk of bias among contributing studies.	N/A

Section and Topic	Item #	Checklist item	Location where item is reported[a]
syntheses	20b	Present results of all statistical syntheses conducted. If meta-analysis was done, present for each the summary estimate and its precision (e.g. confidence/credible interval) and measures of statistical heterogeneity. If comparing groups, describe the direction of the effect.	Page 13–15, Page 18, Table 3, Figs. 2–3, & Suppl. Figs. 2–6
	20c	Present results of all investigations of possible causes of heterogeneity among study results.	Page 18 & Table 2
	20d	Present results of all sensitivity analyses conducted to assess the robustness of the synthesized results.	Page 14–15, Page 17–18, Suppl. Tables 5–7, & Suppl. Fig. 7
Reporting biases	21	Present assessments of risk of bias due to missing results (arising from reporting biases) for each synthesis assessed.	N/A
Certainty of evidence	22	Present assessments of certainty (or confidence) in the body of evidence for each outcome assessed.	N/A
**DISCUSSION**			
Discussion	23a	Provide a general interpretation of the results in the context of other evidence.	Page 18–22
	23b	Discuss any limitations of the evidence included in the review.	Page 22–23
	23c	Discuss any limitations of the review processes used.	Page 22–23
	23d	Discuss implications of the results for practice, policy, and future research.	Page 20–24
**OTHER INFORMATION**			
Registration and protocol	24a	Provide registration information for the review, including register name and registration number, or state that the review was not registered.	Page 5
	24b	Indicate where the review protocol can be accessed, or state that a protocol was not prepared.	Page 5

Section and Topic	Item #	Checklist item	Location where item is reported[a]
	24c	Describe and explain any amendments to information provided at registration or in the protocol.	N/A
Support	25	Describe sources of financial or non-financial support for the review, and the role of the funders or sponsors in the review.	Page 26
Competing interests	26	Declare any competing interests of review authors.	Page 26
Availability of data, code and other materials	27	Report which of the following are publicly available and where they can be found: template data collection forms; data extracted from included studies; data used for all analyses; analytic code; any other materials used in the review.	Page 26

[a]Locations were based on the submitted manuscript file.

N/A, not applicable or not available; **PRISMA**, Preferred Reporting Items for Systematic Reviews and Meta-Analyses.

Supplementary Table 2. PICO framework [2].

Components of PICO	Definition
**P**opulation	Adults aged 18 years or older
**I**ndex Test	LAP
**C**omparator	MetS diagnostic criteria
**O**utcome	Diagnostic accuracy (AUC, sensitivity, and specificity)

AUC, area under curve; **LAP**, lipid accumulation product; **MetS**, metabolic syndrome; **PICO**, Population, Index Test, Comparator, and Outcome.

Supplementary Table 3. Modified QUADAS-2 tool signaling questions for risk of bias [3].

No.	Signaling Questions	Yes	Unclear	No
		**Patient Selection**		
**#1**	**Was a consecutive or random sample of patients enrolled?**	A consecutive or random sample of patients was enrolled.	It is unclear whether a consecutive or random sample of patients was enrolled.	There was no consecutive or random sample of patients enrolled.
**#2**	**Was a case-control design avoided?**	A case-control design was avoided.	It is unclear if a case-control design was avoided.	The study used a case-control design.
**#3**	**Did the study avoid inappropriate exclusions?**	There were no inappropriate exclusions of patients.	It is unclear if there were inappropriate exclusions of patients.	There were inappropriate exclusions of patients (e.g., patients with any physical and postural limitations precluding anthropometric measurements, patients with history of weight loss surgeries or any other procedures that caused WC reduction, such as liposuction, lipolysis, and abdominoplasty).
		**Index Test**		
**#4**	**Were the index test results interpreted without knowledge of the results of the reference standard?**	This item was omitted since blinding LAP interpreter was considered to be irrelevant to the current study. LAP is an objective index test as it involves an objective measurement of TG using machine analyzers and a specific formula to obtain the final value.		
**#5**	**If a threshold was used, was it pre-specified?**	This item was omitted as the specific threshold of LAP for MetS has not been determined to date; hence, each study analyzed its own population optimal cut-off value.		
**#6**	**If a threshold was used, was the sample size included in the diagnostic analysis ≥ 300?[a]**	A threshold was used and the sample size included in the LAP accuracy analysis was ≥ 300.	It is unclear whether a threshold was used or the sample size included in the LAP accuracy analysis was ≥ 300.	A threshold was used and the sample size included in the LAP accuracy analysis was < 300.
**#7**	**Was the calculation to determine the index test clearly described?[b]**	The calculation including formula and units of LAP was clearly described.	One of the calculation components of LAP (formula or units) was not clearly described.	The calculation including formula and units of LAP was not clearly described.
**#8**	**Were the methods used to determine the index test consistently applied across study subjects?[b]**	The methods used to determine LAP were consistently applied across study subjects.	It is unclear if the methods used to determine LAP were consistently applied across study subjects.	The methods used to determine LAP were not consistently applied across study subjects.
		**Reference Standard**		
**#9**	**Is the reference standard likely to correctly classify the target condition?**	The study used published and established criteria to define MetS.	It is unclear what criteria were used to define MetS.	The study did not use any published and established criteria to define MetS.
**#10**	**Were the reference standard results interpreted without knowledge of the results of the index test?**	This item was omitted as blinding the MetS diagnostic criteria interpreter was considered to be irrelevant to the current study. Since the threshold of LAP for MetS has not been determined to date, the MetS diagnostic criteria results would always be interpreted without the knowledge of the results of LAP.		
**#11**	**Was the reference standard and its definition clearly described?[b]**	The type of MetS diagnostic criteria and all of the MetS components definitions were clearly described.	The type of MetS diagnostic criteria was clearly described without complete description of the MetS components.	Either the type of MetS diagnostic criteria or all of the MetS components definitions was not clearly described.

#12	**Were the methods used to determine the reference standard consistently applied across study subjects?[b]**	The methods used to determine MetS were consistently applied across study subjects.	It is unclear if the methods used to determine MetS were consistently applied across study subjects.	The methods used to determine MetS were not consistently applied across study subjects.
		**Flow and Timing**		
#13	**Was there an appropriate interval between index test(s) and reference standard?**	There was an appropriate interval between the result collection of LAP and MetS.	It is not clear whether the interval between the result collection of LAP and MetS was appropriate.	There was no appropriate interval between the result collection of LAP and MetS.
#14	**Did all patients receive a reference standard?**	All patients received the definition criteria of MetS.	It is unclear if all patients received the definition criteria of MetS.	Some patients did not receive the definition criteria of MetS.
#15	**Did patients receive the same reference standard?**	All patients received the same definition criteria of MetS.	It is not clear if all patients received the same definition criteria of MetS.	Some patients received different definition criteria of MetS.
#16	**Were all patients included in the analysis?**	All patients were included in the LAP accuracy analysis.	It is not clear whether all patients were included in the LAP accuracy analysis.	Some patients were not included in the LAP accuracy analysis.

[a]This additional item is formulated from a rule-of-thumb suggested by Bujang et al. [4], where a sample of minimum 300 subjects can often be considered sufficient in obtaining a reliable estimate of the sensitivity and specificity of most screening and diagnostic tests.
[b]These additional items are adopted and formulated from the QUADAS-2 signaling questions in McCrea et al. [5] and Munthali et al. [6].
LAP, lipid accumulation product; **MetS**, metabolic syndrome; **QUADAS-2**, Quality Assessment of Diagnostic Accuracy Studies 2; **WC**, waist circumference.

Supplementary Table 4. Outcomes of the included studies.

Author, Year	Gender	Value of LAP[a]		Association of LAP and MetS			Diagnostic Accuracy Parameters of LAP for MetS							
		**MetS**	**Non-MetS**	**OR (95% CI)**	***p*-value**	**Model**	**AUC (95% CI)**	**Optimal Cut-Off**	**% Sn**	**% Sp**	**TP**	**FP**	**FN**	**TN**
**Adejumo et al., 2019** [7]	Male	42.17 ± 24.72	21.77 ± 20.02	N/A	N/A	N/A	0.773 (0.693–0.852)	22.94	88	66.1	20	42	3	81
	Female	53.95 ± 34.90	27.01 ± 18.40	N/A	N/A	N/A	0.801 (0.748–0.853)	28.29	81.8	61.4	82	112	18	177
**Alfawaz et al., 2023** [8]	Male	78.6 (53.3, 121.9)	29.9 (19.3, 46.1)	N/A	N/A	N/A	0.877 (0.840–0.909)	46.2	85.63	76.26	151	47	25	151
	Female	63.9 (44.5, 93.0)	31.0 (19.3, 45.2)	N/A	N/A	N/A	0.840 (0.803–0.872)	49.82	68.53	82.38	159	40	73	187
**Alves et al., 2021** [9]	Male	N/A	N/A	N/A	N/A	N/A	0.781 (0.676–0.886)	41.36	86.7	64.7	39	12	6	22
	Female	N/A	N/A	N/A	N/A	N/A	0.933 (0.871–0.994)	46.82	85.9	77.8	61	2	10	7
**Anto et al., 2023** [10]	Male	N/A	N/A	N/A	N/A	N/A	0.951 (0.912–0.991)	23.87	95	84	381	224	20	1,176
	Female	N/A	N/A	N/A	N/A	N/A	0.790 (0.718–0.862)	33.32	84	60	1,244	583	237	875
**Banik et al., 2021** [11]	Male (age 40–59 years)	N/A	N/A	1.03	< 0.05	Unadjusted	0.72 (0.57–0.87)	58.83	70.8	66.7	17	7	7	14
	Male (age 60–65 years)	N/A	N/A	1.05	NS	Unadjusted	0.88 (0.71–1.00)	36.41	100	72.7	4	3	0	8
	Female (age 20–39 years)	68.7 (53.8, 91.3)	31.6 (20.7, 44.7)	1.02	< 0.05	Unadjusted	0.79 (0.63–0.94)	50.19	66.7	81.8	8	6	4	28
	Female (age 40–59 years)			1.08	< 0.001	Unadjusted	0.87 (0.81–0.94)	54.01	78.3	85.4	47	6	13	35

	Female (age 60–65 years)			1.14	< 0.05	Unadjusted	0.96 (0.89–1.00)	42.06	95	100	19	0	1	6
**Chiang et al., 2012** [12]	Male	N/A	N/A	1.10 (1.07–1.13)	< 0.001	Unadjusted	0.916 (0.880–0.953)	31.64	88.46	82.24	46	38	6	176
	Female	N/A	N/A	1.12 (1.08–1.15)	< 0.001	Unadjusted	0.901 (0.855–0.946)	26.97	81.13	87.11	43	25	10	169
**Ching et al., 2020** [13]	Male	75.30 ± 35.10	26.78 ± 16.65	N/A	N/A	N/A	0.923 (0.867–0.980)	41.44	85.7	85.3	24	10	4	58
	Female	63.53 ± 52.65	17.38 ± 11.19	N/A	N/A	N/A	0.920 (0.862–0.977)	21.74	94.7	70.5	36	41	2	98
**Duan et al., 2013** [14]	Male	68.5 ± 35.9	24.7 ± 19.6	N/A	N/A	N/A	0.91 (0.89–0.92)	39.18	82.6	86	276	169	58	1,036
	Female	72.3 ± 40.5	23.4 ± 16.3	N/A	N/A	N/A	0.93 (0.91–0.94)	37.89	87.6	85.4	212	124	30	723
**Duan et al., 2021** [15]	Male	N/A	N/A	N/A	N/A	N/A	0.831 (0.795–0.867)	52.03	77.5	75.4	158	72	46	221
	Female	N/A	N/A	N/A	N/A	N/A	0.887 (0.834–0.940)	54.84	77.8	87.3	35	21	10	145
**Duan et al., 2022** [16]	Male	69.12 (53.36, 109.12)	28.71 (17.18, 40.53)	1.063 (1.050–1.075)	< 0.001	Adjusted	0.900 (0.874–0.926)	46.28	87.1	82.7	149	77	22	367
	Female	64.94 (49.14, 87.83)	28.75 (17.29, 41.28)	1.060 (1.048–1.073)	< 0.001	Adjusted	0.882 (0.853–0.911)	47.02	79.1	82.9	106	120	28	583
**Ejike, 2011** [17]	Male	60.28 ± 9.26	43.44 ± 6.86	N/A	N/A	N/A	0.937 (0.000–1.000)	49.62	100	81	3	7	0	30
**Gao et al., 2019** [18]	Male (Yi Nationality)	N/A	N/A	N/A	N/A	N/A	0.913 (0.884–0.942)	39.69	84.9	86.4	101	58	18	370
	Male (Han Nationality)	N/A	N/A	N/A	N/A	N/A	0.908 (0.885–0.926)	33	91.21	76.71	166	153	16	506
	Female (Yi Nationality)	N/A	N/A	N/A	N/A	N/A	0.854 (0.826–0.879)	37.3	80.17	77.48	93	134	23	461
	Female (Han Nationality)	N/A	N/A	N/A	N/A	N/A	0.889 (0.872–0.905)	35.2	87.57	78.08	148	268	21	956

**Gu et al., 2018** [19]	Male	51.71 ± 38.60	18.27 ± 13.53	N/A	N/A	N/A	0.897 (0.885–0.907)	26.35	85.09	79.31	850	430	149	1,648
	Female	55.25 ± 40.15	21.75 ± 13.16	N/A	N/A	N/A	0.875 (0.864–0.886)	31.04	79.17	80.69	1,361	372	358	1,554
**Guo et al., 2016** [20]	Male	N/A	N/A	N/A	N/A	N/A	0.853 (0.840–0.867)	34.7	73.93	83.15	797	558	281	2,753
	Female	N/A	N/A	N/A	N/A	N/A	0.817 (0.804–0.829)	27.3	78.88	69.71	1,173	1,258	314	2,895
**İlhan et al., 2019** [21]	Female	83.52 ± 30.03	41.05 ± 19.89	N/A	N/A	N/A	0.88 (0.82–0.93)	54.09	84	78	53	30	10	107
**Jian et al., 2022** [22]	Male	61.50 ± 47.22	23.09 ± 20.99	N/A	N/A	N/A	0.831 (0.806–0.856)	39.7	66.7	86.3	206	256	103	1,609
	Female	61.08 ± 44.12	22.99 ± 19.82	N/A	N/A	N/A	0.842 (0.820–0.864)	35.065	72.5	82.1	277	315	105	1,444
**Lee et al., 2018**[b] [23]	Female	50.79 (35.45, 69.63)	17.09 (10.68, 26.58)	1.09 (1.09–1.10)	< 0.001	Unadjusted	0.91 (0.90–0.92)	29.62	83.3	82.02	379	626	76	2,855
**Li et al., 2022** [24]	Male	N/A	N/A	1.052 (1.043–1.061)	< 0.001	Adjusted	0.869 (0.850–0.887)	53.3125	80.13	78.52	449	286	111	1,047
	Female	N/A	N/A	1.047 (1.038–1.056)	< 0.001	Adjusted	0.846 (0.827–0.865)	52.4291	81.17	76.77	429	342	100	1,130
**Li et al., 2023** [25]	Male	61.21 ± 44.08	17.47 ± 12.57	N/A	N/A	N/A	0.912 (0.903–0.921)	27.895	83.5	83.6	1,111	493	220	2,516
	Female	62.44 ± 39.15	24.09 ± 13.54	N/A	N/A	N/A	0.876 (0.867–0.885)	35.867	75.4	83.4	1,982	413	647	2,075
**Liu et al., 2017** [26]	Male	N/A	N/A	N/A	N/A	N/A	0.904 (0.871–0.937)	33.78	92	74.5	117	14	10	41
	Female	N/A	N/A	N/A	N/A	N/A		50.62	71.1	90.6	43	5	18	48
**Liu et al., 2021** [27]	Male	N/A	N/A	N/A	N/A	N/A	0.963 (0.912–1.000)	20.1	100	85.2	5	34	0	196
	Female	N/A	N/A	N/A	N/A	N/A	0.931 (0.829–1.000)	13.7	100	75.5	4	51	0	158
**Llinás et al., 2017** [28]	Male	118.80 ± 78.58	28.82 ± 24.49	N/A	N/A	N/A	0.946 (0.943–0.950)	36.04	95	75.7	3,035	7,739	160	24,108

	Female	69.86 ± 49.15	15.77 ± 12.62	N/A	N/A	N/A	0.942 (0.935–0.950)	18.4	96	70.1	1,045	7,520	44	17,632
**Luo et al., 2019** [29]	Male	N/A	N/A	N/A	N/A	N/A	0.83	47.46	74.3	79.3	613	398	212	1,525
	Female	N/A	N/A	N/A	N/A	N/A	0.86	48.08	80.3	76.2	1,473	1,301	361	4,165
**Mosad et al., 2023** [30]	Male	80.3 ± 29.2	28.8 ± 17.6	N/A	N/A	N/A	0.970 (0.948–0.993)	49.42	93.5	90.9	72	7	5	70
	Female	96.9 ± 46.0	32.8 ± 17.9	N/A	N/A	N/A	0.964 (0.945–0.982)	52.23	92.8	85.3	123	20	10	113
**Motamed et al., 2016** [31]	Male	N/A	N/A	N/A	N/A	N/A	0.899 (0.888–0.910)	39.89	86	79.6	958	409	156	1,596
	Female	N/A	N/A	N/A	N/A	N/A	0.915 (0.904–0.927)	49.71	85.2	82.3	827	252	144	1,169
**Musa et al., 2023** [32]	Male	N/A	N/A	N/A	N/A	N/A	0.908 (0.857–0.960)	50	90.9	80	10	25	1	99
	Female	N/A	N/A	N/A	N/A	N/A	0.721 (0.600–0.842)	41.5	92.9	60	13	20	1	31
**Nwankwo et al., 2023[b]** [33]	Male	50.75 ± 18.90	17.81 ± 17.71	N/A	N/A	N/A	0.82 (0.80–0.83)	27	73	79	159	531	59	1,999
	Female	51.49 ± 8.77	23.64 ± 8.52	N/A	N/A	N/A	0.76 (0.73–0.78)	38	57	84	271	151	205	793
**Omuse et al., 2017** [34]	Male	N/A	N/A	N/A	N/A	N/A	0.949 (0.923–0.976)	42.895	91	84.3	58	30	6	161
	Female	N/A	N/A	N/A	N/A	N/A	0.822 (0.764–0.879)	30.56	77.5	72.8	55	55	16	147
**Osman et al., 2020** [35]	Female	103.46 ± 58.22	41.43 ± 23.03	N/A	N/A	N/A	0.895 (0.860–0.930)	56.23	82	80	122	28	27	113
**Rabiei et al., 2021** [36]	Male	73.07 ± 36.07	33.45 ± 19.50	N/A	N/A	N/A	0.87 (0.85–0.89)	49.31	74	83.71	336	116	118	596
	Female	82.37 ± 44.08	40.06 ± 19.30	N/A	N/A	N/A	0.85 (0.82–0.87)	52.39	76.09	76.9	646	94	203	313
	Male	65.1 ± 24.7	31.9 ± 18.8	1.078 (1.024–1.136)	0.004	Adjusted	0.882	45.65	80	80	44	19	11	76

**Rajendran et al., 2022** [37]	Female	63.3 ± 19.7	29.8 ± 15.6	1.149 (1.016–1.299)	0.027	Adjusted	0.905	46.91	88	88	37	13	5	95
**Shabestari et al., 2016** [38]	Female	N/A	N/A	N/A	N/A	N/A	0.827 (0.775–0.879)	47.63	75	77.9	82	34	27	121
**Shao et al., 2023** [39]	Male	N/A	N/A	1.074 (1.071–1.077)	< 0.001	Adjusted	0.901 (0.895–0.906)	36.25	81.91	81.6	3,183	1,933	703	8,572
	Female	N/A	N/A	1.097 (1.094–1.100)	< 0.001	Adjusted	0.898 (0.893–0.902)	34.95	80.93	83.04	5,947	2,325	1,401	11,382
**Shin et al., 2019** [40]	Male	N/A	N/A	N/A	N/A	N/A	0.899 (0.893–0.906)	40.78	83.6	82.8	1,034	1,463	203	7,043
	Female	N/A	N/A	N/A	N/A	N/A	0.953 (0.946–0.960)	23.85	92.6	85.5	603	739	48	4,357
**Soares, 2016** [41]	Male	101.98 ± 93.90	23.84 ± 15.63	N/A	N/A	N/A	0.92 (0.86–0.98)	33.16	89.7	82.9	35	7	4	34
	Female	71.34 ± 50.88	22.95 ± 16.68	N/A	N/A	N/A	0.88 (0.82–0.94)	30.1	85.7	76.4	36	21	6	68
**Su et al., 2020** [42]	Male	N/A	N/A	1.099 (1.075–1.123)	< 0.05	Adjusted	0.831 (0.797–0.862)	39.2	69.4	80.1	129	71	57	285
	Female	N/A	N/A	1.107 (1.086–1.129)	< 0.05	Adjusted	0.865 (0.839–0.888)	43.25	80.7	79.7	221	105	53	413
**Talavera et al., 2022** [43]	Male	99.15 (77.53, 132.24)	25.32 (14.84, 44.01)	N/A	N/A	N/A	0.929 (0.907–0.952)	59.85	91.6	84.5	98	283	9	1,546
	Female	88.01 (68.72, 116.37)	29.32 (19.78, 43.33)	N/A	N/A	N/A	0.950 (0.940–0.960)	53.06	92.4	86.4	462	211	38	1,344
**Taverna et al., 2011** [44]	Male	89.79 ± 46.90	34.65 ± 21.42	N/A	N/A	N/A	0.910 (0.860–0.950)	51.82	85	85	45	45	8	254
	Female	58.79 ± 30.00	21.96 ± 14.17	N/A	N/A	N/A	0.900 (0.860–0.940)	33.28	81	80	52	70	12	282
**Tellechea et al., 2009** [45]	Male	N/A	N/A	N/A	N/A	N/A	0.91	53.63	83	83	131	75	27	368

**Xiang et al., 2012**[b] [46]	Male	N/A	N/A	N/A	N/A	N/A	0.88	44.5	85.3	80.6	189	167	32	694
	Female	N/A	N/A	N/A	N/A	N/A	0.931	37.7	95.8	83	203	206	9	1,004
**Yin et al., 2018** [47]	Male	N/A	N/A	N/A	N/A	N/A	0.907 (0.839–0.888)	39.83	88.2	86.2	47	7	6	43
	Female	N/A	N/A	N/A	N/A	N/A		37.16	87.4	85.5	25	7	4	43
**Zhang et al., 2017** [48]	Male	N/A	N/A	N/A	N/A	N/A	0.852 (0.805–0.898)	32.8	76.5	80.7	80	42	24	174
	Female	N/A	N/A	N/A	N/A	N/A	0.871 (0.837–0.905)	40.4	74.6	86.6	146	35	50	229
**Zhang et al., 2019** [49]	Male	51.71 ± 38.60	18.27 ± 13.53	N/A	N/A	N/A	0.896	26.36	75	84	74	33	25	174
	Female	55.25 ± 40.15	21.75 ± 13.16	N/A	N/A	N/A	0.874	31.05	74	92	127	15	45	176

[a]Data are presented in mean ± SD or median (IQR).

[b]Authors that provided additional data on requests.

AUC, area under curve; **CI**, confidence interval; **FN**, false negative; **FP**, false positive; **IQR**, interquartile range; **LAP**, lipid accumulation product; **MetS**, metabolic syndrome; **N/A**, not applicable or not available; **OR**, odds ratio; **SD**, standard deviation; **Sn**, sensitivity; **Sp**, specificity; **TN**, true negative; **TP**, true positive.

Supplementary Table 5. Sensitivity analyses for MD meta-analyses of LAP in MetS and non-MetS subjects.

Analysis	Male				Female			
	**Number of Studies**	**MD (95% CI)**	***p*-value**	***I*²**	**Number of Studies**	**MD (95% CI)**	***p*-value**	***I*²**
**Main analysis**	18	45.92 (36.11–55.72)	< 0.001	99%	21	41.70 (37.16–46.24)	< 0.001	97%
**Sensitivity analyses**								
**Without outliers**	12	44.02 (40.53–47.51)	< 0.001	74%	13	39.28 (36.98–41.58)	< 0.001	58%
**Low risk of bias**	9	48.32 (33.59–63.05)	< 0.001	99%	9	40.73 (33.65–47.81)	< 0.001	99%
**Sample size ≥ 100**	15	46.19 (35.60–56.78)	< 0.001	99%	21	41.70 (37.16–46.24)	< 0.001	97%

CI, confidence interval; **LAP**, lipid accumulation product; **MD**, mean difference; **MetS**, metabolic syndrome.

Supplementary Table 6. Sensitivity analyses for OR meta-analyses between LAP and MetS.

Analysis	Male				Female			
	Number of Studies	OR (95% CI)	*p*-value	I^2	Number of Studies	OR (95% CI)	*p*-value	I^2
**Main analysis**	6	1.07 (1.06–1.09)	< 0.001	83%	7	1.08 (1.07–1.10)	< 0.001	95%
**Sensitivity analyses**								
**Without outliers**	6	1.07 (1.06–1.09)	< 0.001	83%	6	1.09 (1.08–1.10)	< 0.001	87%
**Low risk of bias**	5	1.07 (1.06–1.08)	< 0.001	84%	5	1.08 (1.05–1.11)	< 0.001	97%
**Sample size ≥ 100**	6	1.07 (1.06–1.09)	< 0.001	83%	7	1.08 (1.07–1.10)	< 0.001	95%

CI, confidence interval; **LAP**, lipid accumulation product; **MetS**, metabolic syndrome; **OR**, odds ratio.

Supplementary Table 7. Sensitivity analyses for diagnostic accuracy meta-analyses of LAP as a screening tool for MetS.

**Analysis**	**Male**						**Female**					
	**Number of Studies**	**AUSROC Curve (95% CI)**	**% Sn (95% CI)**	**% Sp (95% CI)**	***I^2* of Sn**	***I^2* of Sp**	**Number of Studies**	**AUSROC Curve (95% CI)**	**% Sn (95% CI)**	**% Sp (95% CI)**	***I^2* of Sn**	***I^2* of Sp**
**Main analysis**	39	0.88 (0.85–0.90)	85 (82–87)	81 (80–83)	95%	95%	41	0.88 (0.85–0.91)	83 (80–86)	80 (78–82)	94%	98%
**Sensitivity analyses**												
**Without outliers**	33	0.87 (0.84–0.90)	84 (81–86)	82 (81–83)	87%	76%	31	0.88 (0.85–0.91)	82 (80–84)	81 (80–83)	88%	95%
**Low risk of bias**	16	0.87 (0.84–0.90)	85 (80–88)	82 (80–83)	97%	98%	16	0.88 (0.85–0.91)	82 (77–87)	81 (78–84)	97%	99%
**Sample size ≥ 100**	34	0.88 (0.85–0.90)	84 (82–87)	82 (80–83)	95%	96%	37	0.88 (0.85–0.91)	83 (80–85)	81 (78–83)	94%	98%

AUSROC, area under the summary receiver operating characteristic; **CI**, confidence interval; **LAP**, lipid accumulation product; **MetS**, metabolic syndrome; **Sn**, sensitivity; **Sp**, specificity.

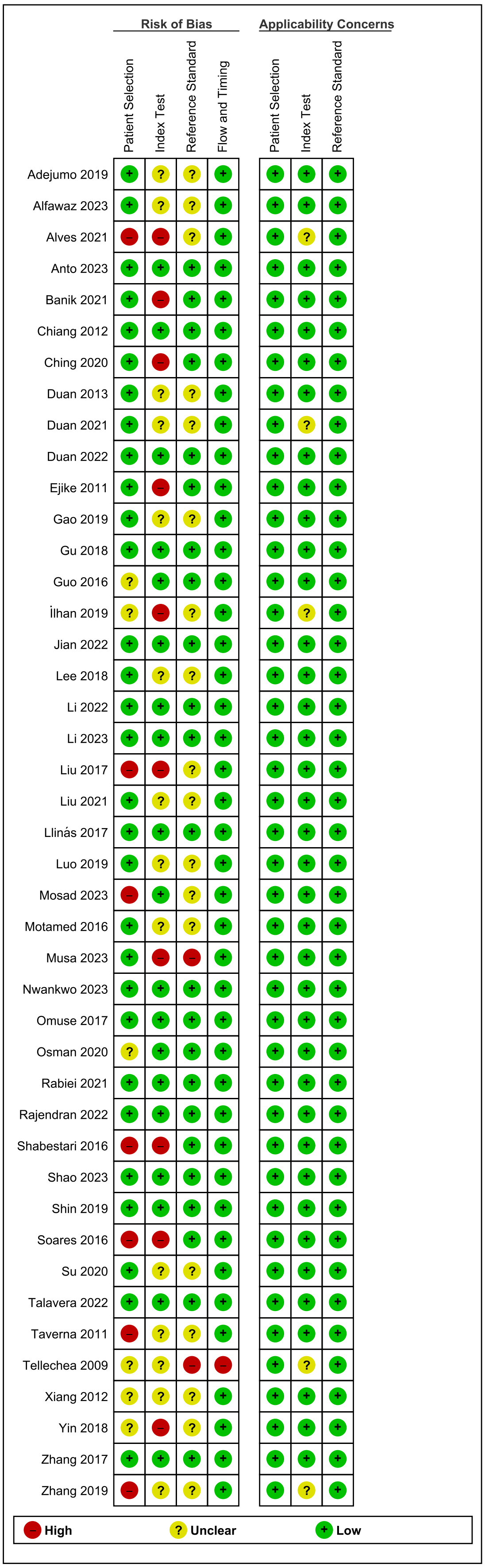

Supplementary Fig. 1. Domain-specific quality assessment results of included studies using the QUADAS-2 tool [3]. **QUADAS-2**, Quality Assessment of Diagnostic Accuracy Studies 2.

A

Study or Subgroup	MetS Mean	MetS SD	MetS Total	Non-MetS Mean	Non-MetS SD	Non-MetS Total	Weight	Mean Difference IV, Random, 95% CI
Adejumo 2019	42.17	24.72	23	21.77	20.02	123	5.5%	20.40 [9.70, 31.10]
Alfawaz 2023	84.92	51.28	176	31.87	20.01	198	5.6%	53.05 [44.98, 61.12]
Ching 2020	75.3	35.1	28	26.78	16.65	68	5.3%	48.52 [34.93, 62.11]
Duan 2013	68.5	35.9	334	24.7	19.6	1205	5.8%	43.80 [39.79, 47.81]
Duan 2022	77.63	41.69	171	28.81	17.37	444	5.7%	48.82 [42.37, 55.27]
Ejike 2011	60.28	9.26	3	43.44	6.86	37	5.5%	16.84 [6.13, 27.55]
Gu 2018	51.71	38.6	999	18.27	13.53	2078	5.8%	33.44 [30.98, 35.90]
Jian 2022	61.5	47.22	309	23.09	20.99	1865	5.8%	38.41 [33.06, 43.76]
Li 2023	61.21	44.08	1331	17.47	12.57	3009	5.8%	43.74 [41.33, 46.15]
Llinás 2017	118.8	78.58	3195	28.82	24.49	31847	5.8%	89.98 [87.24, 92.72]
Mosad 2023	80.3	29.2	77	28.8	17.6	77	5.7%	51.50 [43.88, 59.12]
Nwankwo 2023	50.75	18.9	218	17.81	17.71	2530	5.8%	32.94 [30.34, 35.54]
Rabiei 2021	73.07	36.07	454	33.45	19.5	712	5.8%	39.62 [36.01, 43.23]
Rajendran 2022	65.1	24.7	55	31.9	18.8	95	5.7%	33.20 [25.66, 40.74]
Soares 2016	101.98	93.9	39	23.84	15.63	41	3.8%	78.14 [48.28, 108.00]
Talavera 2022	103.19	41.11	107	28.19	21.64	1829	5.6%	75.00 [67.15, 82.85]
Taverna 2011	89.79	46.9	53	34.65	21.42	299	5.3%	55.14 [42.28, 68.00]
Zhang 2019	51.71	38.6	99	18.27	13.53	207	5.6%	33.44 [25.62, 41.26]
**Total (95% CI)**			**7671**			**46664**	**100.0%**	**45.92 [36.11, 55.72]**

Heterogeneity: Tau² = 427.07; Chi² = 1346.90, df = 17 (P < 0.00001); I² = 99%
Test for overall effect: Z = 9.18 (P < 0.00001)

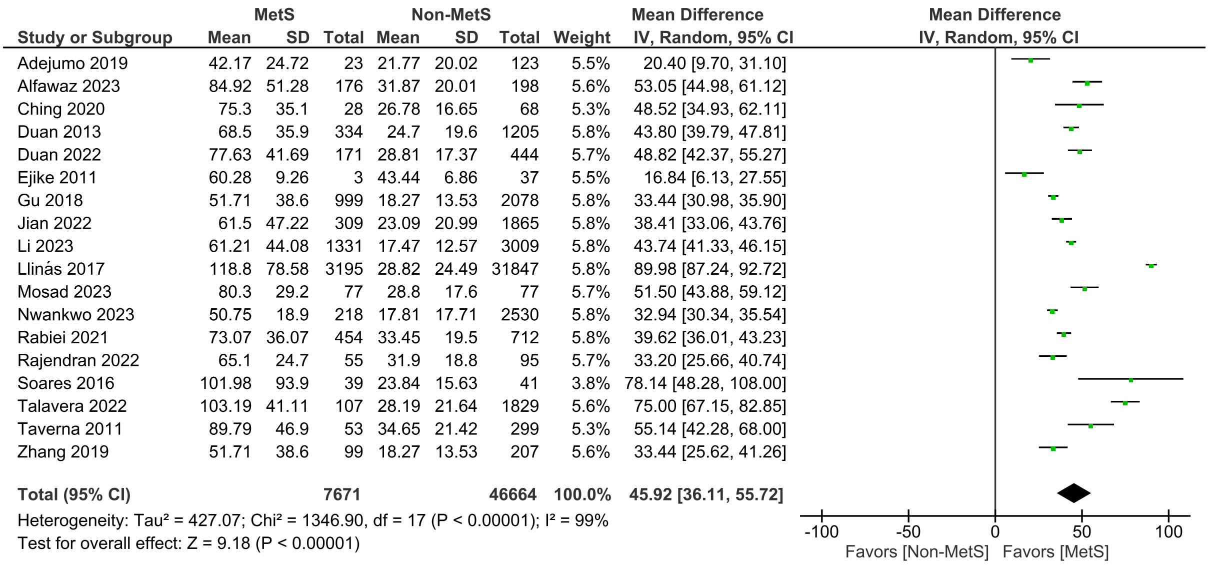

B

Study or Subgroup	MetS Mean	MetS SD	MetS Total	Non-MetS Mean	Non-MetS SD	Non-MetS Total	Weight	Mean Difference IV, Random, 95% CI
Adejumo 2019	53.95	34.9	100	27.01	18.4	289	4.7%	26.94 [19.78, 34.10]
Alfawaz 2023	67.3	36.18	232	31.88	19.32	227	5.0%	35.42 [30.13, 40.71]
Banik 2021	71.41	28.24	92	32.38	18.12	81	4.7%	39.03 [32.04, 46.02]
Ching 2020	63.53	52.65	38	17.38	11.19	139	3.1%	46.15 [29.31, 62.99]
Duan 2013	72.3	40.5	242	23.4	16.3	847	5.0%	48.90 [43.68, 54.12]
Duan 2022	67.43	29	134	29.12	17.82	703	5.0%	38.31 [33.23, 43.39]
Gu 2018	55.25	40.15	1719	21.75	13.16	1926	5.3%	33.50 [31.51, 35.49]
İlhan 2019	83.52	30.03	63	41.05	19.89	137	4.6%	42.47 [34.34, 50.60]
Jian 2022	61.08	44.12	382	22.99	19.82	1759	5.1%	38.09 [33.57, 42.61]
Lee 2018	52.02	25.42	455	18.17	11.79	3481	5.3%	33.85 [31.48, 36.22]
Li 2023	62.44	39.15	2629	24.09	13.54	2488	5.3%	38.35 [36.76, 39.94]
Llinás 2017	69.86	49.15	1089	15.77	12.62	25152	5.2%	54.09 [51.17, 57.01]
Mosad 2023	96.9	46	133	32.8	17.9	133	4.5%	64.10 [55.71, 72.49]
Nwankwo 2023	51.49	8.77	476	23.64	8.52	944	5.3%	27.85 [26.89, 28.81]
Osman 2020	103.46	58.22	149	41.43	23.03	141	4.2%	62.03 [51.94, 72.12]
Rabiei 2021	82.37	44.08	849	40.06	19.3	407	5.2%	42.31 [38.80, 45.82]
Rajendran 2022	63.3	19.7	42	29.8	15.6	108	4.8%	33.50 [26.86, 40.14]
Soares 2016	71.34	50.88	42	22.95	16.68	89	3.2%	48.39 [32.62, 64.16]
Talavera 2022	91.19	35.43	500	30.89	17.47	1555	5.2%	60.30 [57.08, 63.52]
Taverna 2011	58.79	30	64	21.96	14.17	352	4.7%	36.83 [29.33, 44.33]
Zhang 2019	55.25	40.15	172	21.75	13.16	191	4.8%	33.50 [27.22, 39.78]
**Total (95% CI)**			**9602**			**41149**	**100.0%**	**41.70 [37.16, 46.24]**

Heterogeneity: Tau² = 100.73; Chi² = 770.40, df = 20 (P < 0.00001); I² = 97%
Test for overall effect: Z = 18.00 (P < 0.00001)

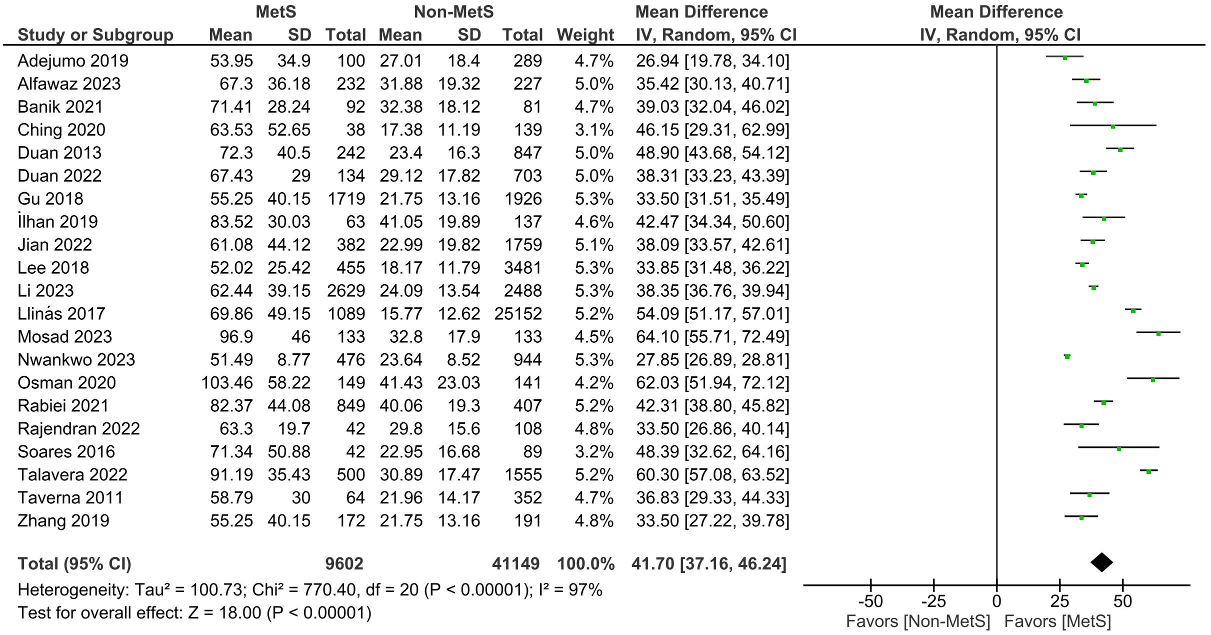

Supplementary Fig. 2. Forest plots of MD meta-analyses of LAP in (**A**) men and (**B**) women with and without MetS. **CI**, confidence interval; **IV**, inverse variance; **LAP**, lipid accumulation product; **MD**, mean difference; **MetS**, metabolic syndrome; **SD**, standard deviation.

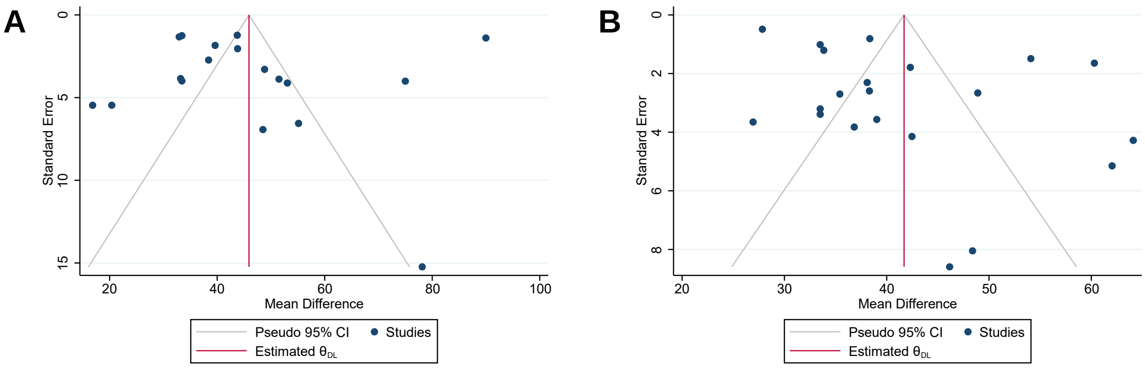

Supplementary Fig. 3. Funnel plots of MD meta-analyses of LAP in (**A**) men and (**B**) women with and without MetS. **CI**, confidence interval; **LAP**, lipid accumulation product; **MD**, mean difference; **MetS**, metabolic syndrome.

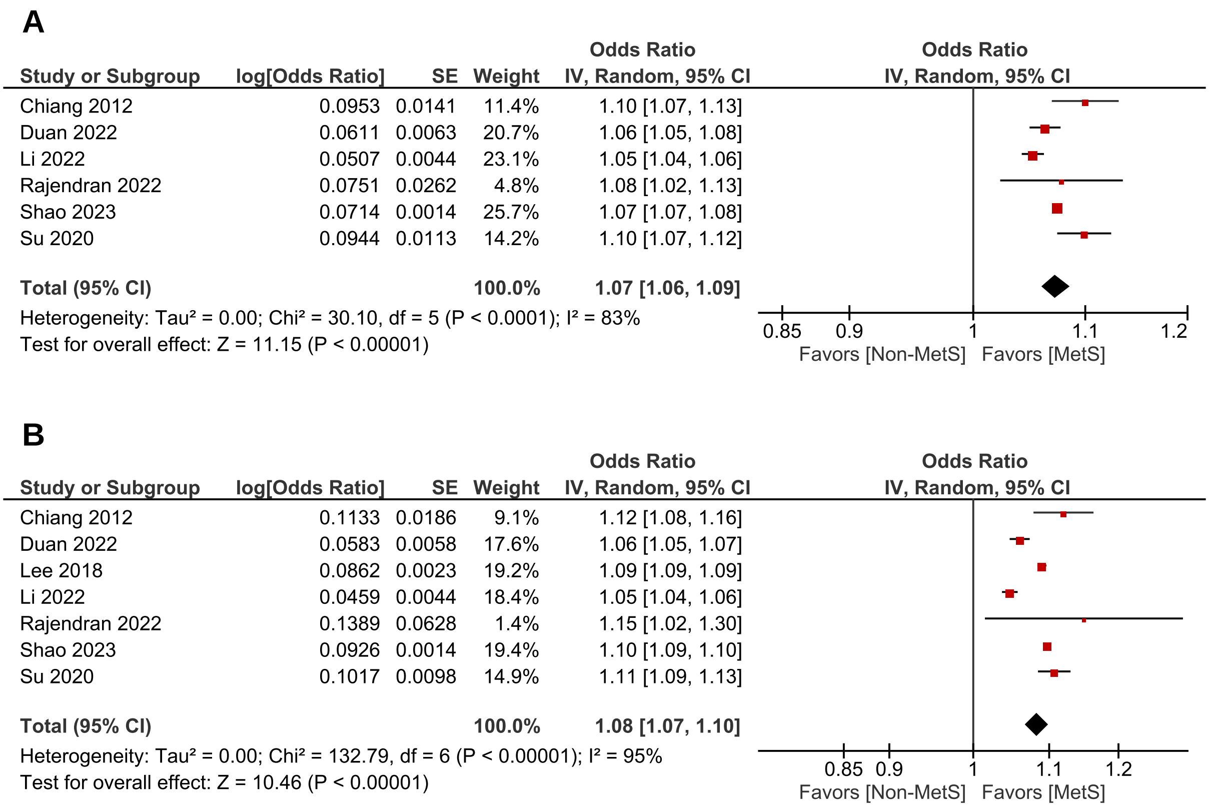

Supplementary Fig. 4. Forest plots of OR meta-analyses between LAP and MetS in (**A**) men and (**B**) women. **CI**, confidence interval; **IV**, inverse variance; **LAP**, lipid accumulation product; **MetS**, metabolic syndrome; **OR**, odds ratio; **SE**, standard error.

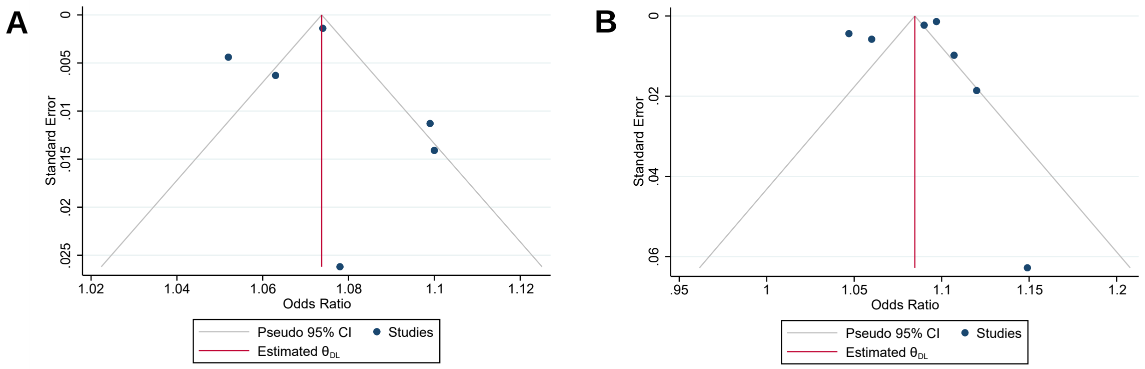

Supplementary Fig. 5. Funnel plots of OR meta-analyses between LAP and MetS in (**A**) men and (**B**) women. **CI**, confidence interval; **LAP**, lipid accumulation product; **MetS**, metabolic syndrome; **OR**, odds ratio.

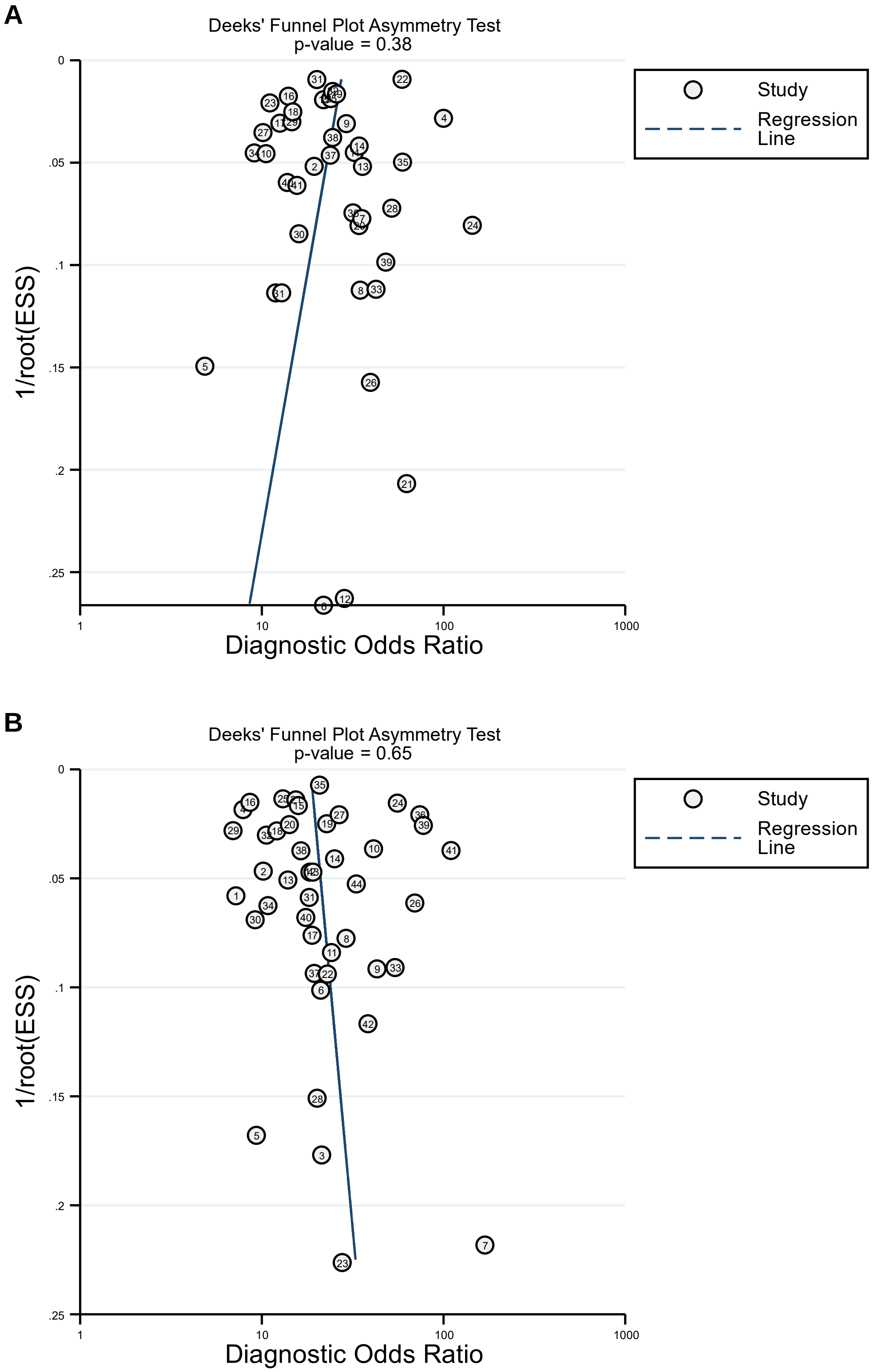

Supplementary Fig. 6. Deeks' funnel plots of diagnostic accuracy meta-analyses of LAP for detecting MetS in (**A**) men and (**B**) women. **ESS**, effective sample size; **LAP**, lipid accumulation product; **MetS**, metabolic syndrome.

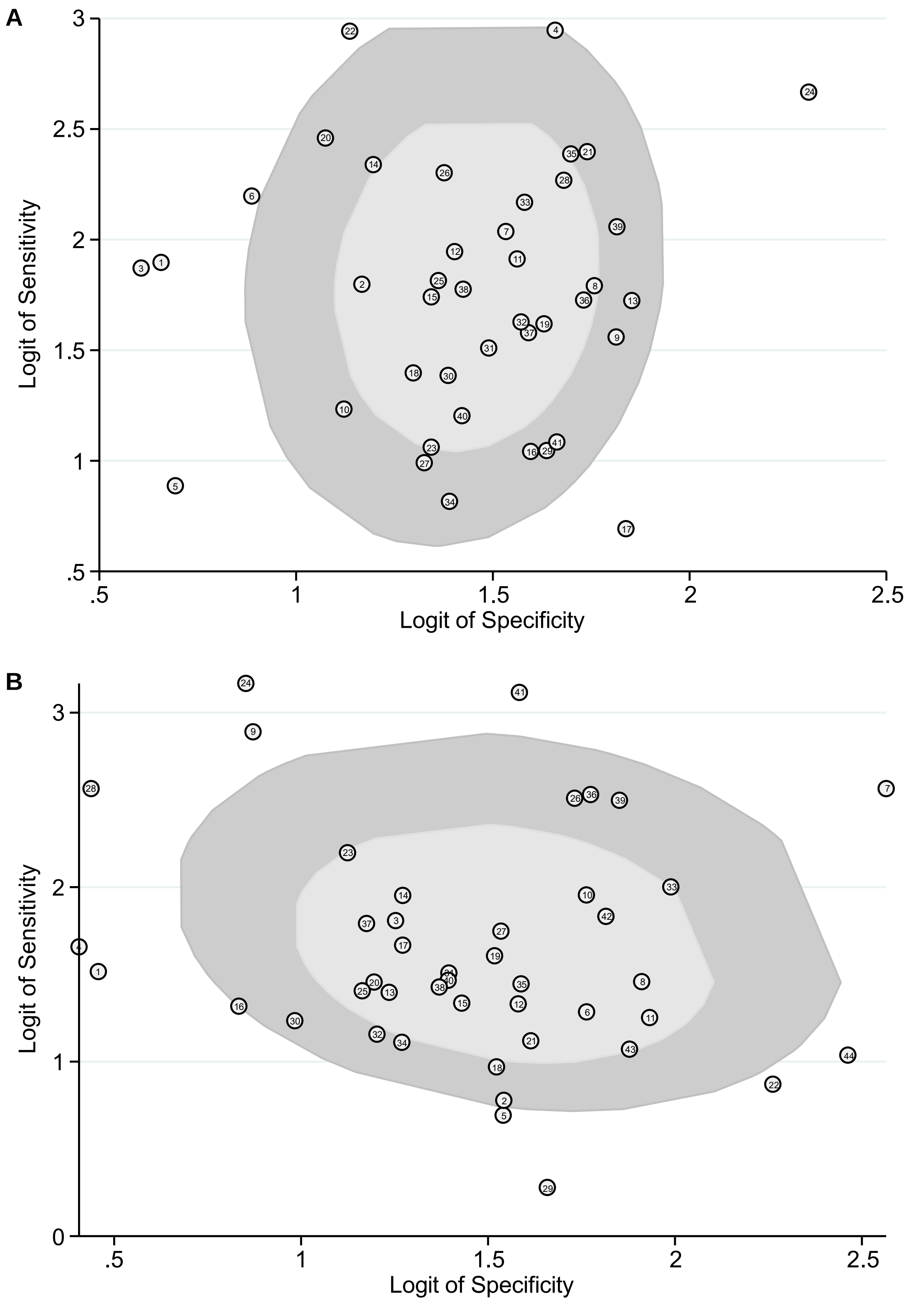

Supplementary Fig. 7. Bivariate boxplots of diagnostic accuracy meta-analyses of LAP for detecting MetS in (**A**) men and (**B**) women. **LAP**, lipid accumulation product; **MetS**, metabolic syndrome.

REFERENCES

[1] Page MJ, McKenzie JE, Bossuyt PM, Boutron I, Hoffmann TC, Mulrow CD, et al. The PRISMA 2020 statement: An updated guideline for reporting systematic reviews. BMJ 2021;372. https://doi.org/10.1136/bmj.n71.

[2] Kim KW, Lee J, Choi SH, Huh J, Park SH. Systematic review and meta-analysis of studies evaluating diagnostic test accuracy: A practical review for clinical researchers–part I. general guidance and tips. Korean J Radiol 2015;16:1175–87. https://doi.org/10.3348/kjr.2015.16.6.1175.

[3] Whiting PF, Rutjes AWS, Westwood ME, Mallett S, Deeks JJ, Reitsma JB, et al. QUADAS-2: A Revised Tool for the Quality Assessment of Diagnostic Accuracy Studies. Ann Intern Med 2011;155:529–36.

[4] Bujang MA, Adnan TH. Requirements for Minimum Sample Size for Sensitivity and Specificity Analysis. J Clin Diagn Res 2016;10:YE01–6. https://doi.org/10.7860/JCDR/2016/18129.8744.

[5] McCrea M, Meier T, Huber D, Ptito A, Bigler E, Debert CT, et al. Role of advanced neuroimaging, fluid biomarkers and genetic testing in the assessment of sport-related concussion: a systematic review. Br J Sports Med 2017;51:919–29. https://doi.org/10.1136/bjsports-2016-097447.

[6] Munthali C, Taegtmeyer M, Garner PG, Lalloo DG, Squire SB, Corbett EL, et al. Diagnostic accuracy of the WHO clinical staging system for defining eligibility for ART in sub-Saharan Africa: a systematic review and meta-analysis. J Int AIDS Soc 2014;17:18932. https://doi.org/10.7448/IAS.17.1.18932.

[7] Adejumo EN, Adejumo AO, Azenabor A, Ekun AO, Enitan SS, Adebola OK, et al. Anthropometric parameter that best predict metabolic syndrome in South west Nigeria. Diabetes Metab Syndr Clin Res Rev 2019;13:48–54. https://doi.org/10.1016/j.dsx.2018.08.009.

[8] Alfawaz HA, Khan N, Ansari MGA, Khattak MNK, Saadawy GM, Al-Daghri NM. Sex-Specific Cut-Offs of Seven Adiposity Indicators and Their Performance in Predicting Metabolic Syndrome in Arab Adults. J Clin Med 2023;12. https://doi.org/10.3390/jcm12237280.

[9] Alves LF, Cruz JO, da Costa Souza AL, de Oliveira CC. Performance of adiposity indicators in predicting metabolic syndrome in older adults. Arch Endocrinol Metab 2021;65:588–95. https://doi.org/10.20945/2359-3997000000372.

[10] Anto EO, Frimpong J, Boadu WIO, Korsah EE, Tamakloe VCKT, Ansah E, et al. Cardiometabolic syndrome among general adult population in Ghana: The role of lipid accumulation product, waist circumference-triglyceride index, and triglyceride-glucose index as surrogate indicators. Heal Sci Reports 2023;6:e1419. https://doi.org/10.1002/hsr2.1419.

[11] Banik SD, Pacheco-Pantoja E, Lugo R, Gómez-De-regil L, Aké RC, González RMM, et al. Evaluation of anthropometric indices and lipid parameters to predict metabolic syndrome among adults in Mexico. Diabetes, Metab Syndr Obes Targets Ther 2021;14:691–701. https://doi.org/10.2147/DMSO.S281894.

[12] Chiang JK, Koo M. Lipid accumulation product: a simple and accurate index for predicting metabolic syndrome in Taiwanese people aged 50 and over. BMC Cardiovasc Disord 2012;12:1. https://doi.org/10.1186/1471-2261-12-78.

[13] Ching YK, Chin YS, Appukutty M, Gan WY, Chan YM. Comparisons of conventional and novel anthropometric obesity indices to predict metabolic syndrome among vegetarians in Malaysia. Sci Rep 2020;10:20861. https://doi.org/10.1038/s41598-020-78035-5.

[14] Duan FY, Li R, Zhang SH, Ren W, Wang ZH, Gong LL, et al. Lipid accumulation product as an effective marker for metabolic syndrome. Chinese J Pract Intern Med 2013;33:552–5.

[15] Duan SJ, Liu ZJ, Chen JL, Yao SK. Predictive Value of Lipid Accumulation Product and Visceral Fat Index for Adult Metabolic Syndrome. Chinese Gen Pract 2021;24:4211–7. https://doi.org/10.12114/j.issn.1007-9572.2021.02.038.

[16] Duan Y, Zhang W, Li Z, Niu Y, Chen Y, Liu X, et al. Predictive ability of obesity- and lipid-related indicators for metabolic syndrome in relatively healthy Chinese adults. Front Endocrinol (Lausanne) 2022;13:1016581. https://doi.org/10.3389/fendo.2022.1016581.

[17] Ejike CECC. Lipid Accumulation Product and Waist-To-Height Ratio Are Predictors of

the Metabolic Syndrome in a Nigerian Male Geriatric Population. J Rural Trop Public Heal 2011;10:101–5.

[18] Gao Y-Y, Feng X-B, Cheng Y-F, Gao Y, Tian H-M, Ren Y, et al. Predictive Value of Obesity Indicators for Metabolic Syndrome in Adults of Han and Yi Nationalities in Sichuan. J Sichuan Univ Med Sci Ed 2019;50:77–82.

[19] Gu Z, Zhu P, Wang Q, He H, Xu J, Zhang L, et al. Obesity and lipid-related parameters for predicting metabolic syndrome in Chinese elderly population. Lipids Health Dis 2018;17:1–8. https://doi.org/10.1186/s12944-018-0927-x.

[20] Guo SX, Zhang XH, Zhang JY, He J, Yan YZ, Ma JL, et al. Visceral Adiposity and Anthropometric Indicators as Screening Tools of Metabolic Syndrome among Low Income Rural Adults in Xinjiang. Sci Rep 2016;6:1–8. https://doi.org/10.1038/srep36091.

[21] İlhan GA, Yıldızhan B. Visceral adiposity indicators as predictors of metabolic syndrome in postmenopausal women. Turkish J Obstet Gynecol 2019;16:164–8. https://doi.org/10.4274/tjod.galenos.2019.62558.

[22] Jian L-Y, Guo S-X, Ma R-L, He J, Rui D-S, Ding Y-S, et al. Comparison of obesity-related indicators for identifying metabolic syndrome among normal-weight adults in rural Xinjiang, China. BMC Public Health 2022;22:1730. https://doi.org/10.1186/s12889-022-14122-8.

[23] Lee HJ, Jo HN, Kim YH, Kim SC, Joo JK, Lee KS. Predictive value of lipid accumulation product, fatty liver index, visceral adiposity index for metabolic syndrome according to menopausal status. Metab Syndr Relat Disord 2018;16:477–82. https://doi.org/10.1089/met.2018.0019.

[24] Li Y, Zheng R, Li S, Cai R, Ni F, Zheng H, et al. Association Between Four Anthropometric Indexes and Metabolic Syndrome in US Adults. Front Endocrinol (Lausanne) 2022;13:889785. https://doi.org/10.3389/fendo.2022.889785.

[25] Li Y, Gui J, Liu H, Guo L-L, Li J, Lei Y, et al. Predicting metabolic syndrome by obesity- and lipid-related indices in mid-aged and elderly Chinese: a population-based cross-sectional study. Front Endocrinol (Lausanne) 2023;14:1201132. https://doi.org/10.3389/fendo.2023.1201132.

[26] Liu C, Zhang X, Tang F, Zhang L. Study on the value of lipid accumulation product and visceral adiposity index in the evaluation of metabolic syndrome in the elderly. Chinese J Difficult Complicat Cases 2017;16:60–3. https://doi.org/10.3969/j.issn.1671-6450.2017.01.015.

[27] Liu X, Ma C, Yin F, Wang R, Lu Q, Lu N, et al. Performance of Two Novel Obesity Indicators for the Management of Metabolic Syndrome in Young Adults. Front Endocrinol (Lausanne) 2021;12:10–3. https://doi.org/10.3389/fendo.2021.719416.

[28] Llinás MG, Janer PE, Agudo SG, Casquero RG, González IC. Usefulness in nursing of different anthropometric and analytical indices to assess the existence of metabolic syndrome with the NCEP ATP III and IDF criteria in Spanish Mediterranean population. Med Balear 2017;32:26–34. https://doi.org/10.3306/MEDICINABALEAR.32.01.26.

[29] Luo L, Niu M, Zhang N, Gao Z. Value of lipid accumulation product in screening of metabolic syndrome in middle-aged and elderly people. Clin Focus 2019;34:231–6. https://doi.org/10.3969/j.issn.1004-583X.2019.03.007.

[30] Mosad AS, Elfadil GA, Elhassan SH, Elbashir ZA, S A Husain NEO, Karar T, et al. Diagnostic performance using obesity and lipid-related indices and atherogenic index of plasma to predict metabolic syndrome in the adult sudanese population. Niger J Clin Pract 2023;26:617–24. https://doi.org/10.4103/njcp.njcp_692_22.

[31] Motamed N, Razmjou S, Hemmasi G, Maadi M, Zamani F. Lipid accumulation product and metabolic syndrome: A population-based study in northern Iran, Amol. J Endocrinol Invest 2016;39:375–82. https://doi.org/10.1007/s40618-015-0369-5.

[32] Musa AH, Ijagila IN, Dungus MM. Evaluation of Metabolic Syndrome Using Lipid Accumulation Products, Visceral Adiposity Index and Body Mass Index in Apparently Healthy Students of University of Maiduguri. East African Sch J Med Sci 2023;6:100–7. https://doi.org/10.36349/easms.2023.v06i03.005.

[33] Nwankwo M, Okamkpa JC, Danborno B, Opoola FO. Anthropometric cut-offs for screening metabolic syndrome in a Nigerian population in southeast Nigeria. J Cardiovasc Dis Res 2023;14:282–99.

[34] Omuse G, Maina D, Hoffman M, Mwangi J, Wambua C, Kagotho E, et al. Metabolic syndrome and its predictors in an urban population in Kenya: A cross sectional study.

BMC Endocr Disord 2017;17:37. https://doi.org/10.1186/s12902-017-0188-0.

[35] Osman A, Dafalla S. Adiposity indices as predictors for metabolic syndrome in postmenopausal women. Natl J Physiol Pharm Pharmacol 2020;10:374–8. https://doi.org/10.5455/njppp.2020.10.01002202007032020.

[36] Rabiei N, Heshmat R, Gharibzadeh S, Ostovar A, Maleki V, Sadeghian M, et al. Comparison of anthro-metabolic indicators for predicting the risk of metabolic syndrome in the elderly population: Bushehr Elderly Health (BEH) program. J Diabetes Metab Disord 2021;20:1439–47. https://doi.org/10.1007/s40200-021-00882-4.

[37] Rajendran S, Kizhakkayil Padikkal AK, Mishra S, Madhavanpillai M. Association of Lipid Accumulation Product and Triglyceride-Glucose Index with Metabolic Syndrome in Young Adults: A Cross-sectional Study. Int J Endocrinol Metab 2022;20:e115428. https://doi.org/10.5812/ijem-115428.

[38] Shabestari AN, Asadi M, Jouyandeh Z, Qorbani M, Kelishadi R. Association of lipid accumulation product with cardio-metabolic risk factors in postmenopausal women. Acta Med Iran 2016;54:370–5.

[39] Shao Q, Li J, Wu Y, Liu X, Wang N, Jiang Y, et al. Enhanced Predictive Value of Lipid Accumulation Product for Identifying Metabolic Syndrome in the General Population of China. Nutrients 2023;15. https://doi.org/10.3390/nu15143168.

[40] Shin KA, Kim YJ. Usefulness of surrogate markers of body fat distribution for predicting metabolic syndrome in middle-aged and older Korean populations. Diabetes, Metab Syndr Obes Targets Ther 2019;12:2251–9. https://doi.org/10.2147/DMSO.S217628.

[41] Soares LM. Produto de acumulação lipídica : acurácia para identificação de portadores da síndrome metabólica em adultos. Dissertation, Brazil, Universidade Federal de Minas Gerais 2016. https://repositorio.ufmg.br/handle/1843/BUBD-AM7NP8. Accessed 20 Jan 2024.

[42] Su ZZ, Li B, Lyu C, Li BB, Wu YY, Wang PX. Predictive value of obesity index for metabolic syndrome in elderly residents in Henan Province. Occup Heal 2020;36:3238–42. https://doi.org/10.13329/j.cnki.zyyjk.2020.0843.

[43] Talavera JE, Torres-Malca JR. Diagnostic performance of lipid accumulation indices

and triglyceride and glucose index for metabolic syndrome in a sample of Peruvian adult population. Rev La Fac Med Humana 2022;22:42–9. https://doi.org/10.25176/RFMH.v22i1.4104.

[44] Taverna MJ, Martínez-Larrad MT, Frechtel GD, Serrano-Ríos M. Lipid accumulation product: A powerful marker of metabolic syndrome in healthy population. Eur J Endocrinol 2011;164:559–67. https://doi.org/10.1530/EJE-10-1039.

[45] Tellechea ML, Aranguren F, Martínez-Larrad MT, Serrano-Ríos M, Taverna MJ, Frechtel GD. Ability of lipid accumulation product to identify metabolic syndrome in healthy men from Buenos Aires. Diabetes Care 2009;32:2008. https://doi.org/10.2337/dc08-2284.

[46] Xiang SK, Hua F, Ren JR, Tang Y, Jiang XH. Diagnostic value of lipid accumulation product in metabolic syndrome in adults. New Med 2012;43:772–5. https://doi.org/10.3969/g.issn.0253-9802.2012.11.005.

[47] Yin L, Fan H, Dong Q, Yu J. Application of LAP and VAI in the diagnosis of adult metabolic syndrome. Chinese J Conval Med 2018;27:1136–8. https://doi.org/10.13517/j.cnki.ccm.2018.11.005.

[48] Zhang Q, Chen X, Shi D, Wang S. Correlation between Different Obesity Measurement Indexes and Risk of Metabolic Syndrome in Middle and Old Aged People in Chengdu. Sichuan Med J 2017;38:16–20. https://doi.org/10.16252/j.cnki.issn1004-0501-2017.01.006.

[49] Zhang N, Wu F, Sun B, Liu XY, Zheng GL. Characteristics of Obesity and Lipid Metabolism-related Parameters in the Patients with Metabolic Syndrome and Their Diagnostic Value. Prog Mod Biomed 2019;19:4192–6. https://doi.org/10.13241/j.cnki.pmb.2019.21.044.
